# Psychometric Characteristics of the Patient-Reported Outcome Measures Applied in the CENTER-TBI Study

**DOI:** 10.3390/jcm10112396

**Published:** 2021-05-28

**Authors:** Nicole von Steinbuechel, Katrin Rauen, Fabian Bockhop, Amra Covic, Ugne Krenz, Anne Marie Plass, Katrin Cunitz, Suzanne Polinder, Lindsay Wilson, Ewout W. Steyerberg, Andrew I. R. Maas, David Menon, Yi-Jhen Wu, Marina Zeldovich, the CENTER-TBI Participants and Investigators

**Affiliations:** 1 Institute of Medical Psychology and Medical Sociology, University Medical Center Göttingen, Waldweg 37A, 37073 Göttingen, Germany; fabian.bockhop@med.uni-goettingen.de (F.B.); amra.covic@med.uni-goettingen.de (A.C.); ugne.krenz@med.uni-goettingen.de (U.K.); annemarie.plass@med.uni-goettingen.de (A.M.P.); katrin.cunitz@med.uni-goettingen.de (K.C.); yi-jhen.wu@med.uni-goettingen.de (Y.-J.W.); marina.zeldovich@med.uni-goettingen.de (M.Z.); 2 Department of Geriatric Psychiatry, Psychiatric Hospital Zurich, University of Zurich, Minervastrasse 145, 8032 Zurich, Switzerland; katrin.rauen@uzh.ch or; 3 Institute for Stroke and Dementia Research (ISD), University Hospital, LMU Munich, Feodor-Lynen-Straße 17, 81377 Munich, Germany; 4 Department of Public Health, Erasmus MC, University Medical Center Rotterdam, 3000 CA Rotterdam, The Netherlands; s.polinder@erasmusmc.nl (S.P.); e.steyerberg@erasmusmc.nl (E.W.S.); 5 Department of Psychology, University of Stirling, Stirling FK9 4LJ, UK; l.wilson@stir.ac.uk; 6 Department of Biomedical Data Sciences, Leiden University Medical Center, 2333 RC Leiden, The Netherlands; 7 Department of Neurosurgery, Antwerp University Hospital and University of Antwerp, 2650 Edegem, Belgium; andrew.maas@uza.be; 8 Division of Anaesthesia, University of Cambridge/Addenbrooke’s Hospital, Box 157, Cambridge CB2 0QQ, UK; dkm13@cam.ac.uk

**Keywords:** psychometric properties, patient-reported outcome measures, traumatic brain injury, classical test theory

## Abstract

Traumatic brain injury (TBI) may lead to impairments in various outcome domains. Since most instruments assessing these are only available in a limited number of languages, psychometrically validated translations are important for research and clinical practice. Thus, our aim was to investigate the psychometric properties of the patient-reported outcome measures (PROM) applied in the CENTER-TBI study. The study sample comprised individuals who filled in the six-months assessments (GAD-7, PHQ-9, PCL-5, RPQ, QOLIBRI/-OS, SF-36v2/-12v2). Classical psychometric characteristics were investigated and compared with those of the original English versions. The reliability was satisfactory to excellent; the instruments were comparable to each other and to the original versions. Validity analyses demonstrated medium to high correlations with well-established measures. The original factor structure was replicated by all the translations, except for the RPQ, SF-36v2/-12v2 and some language samples for the PCL-5, most probably due to the factor structure of the original instruments. The translation of one to two items of the PHQ-9, RPQ, PCL-5, and QOLIBRI in three languages could be improved in the future to enhance scoring and application at the individual level. Researchers and clinicians now have access to reliable and valid instruments to improve outcome assessment after TBI in national and international health care.

## 1. Introduction

Traumatic brain injury (TBI) causes alterations in brain function, as a result of an external force [1], for example, due to falls, road traffic accidents, sports, assaults, or violence. It is a considerable source of disability and death worldwide. The sequelae of TBI not only impact the lives of those affected and their relatives on many different levels [2], but they can also result in high direct and indirect costs [3,4].

Concerning the global prevalence of TBI, the vast majority of individuals experience mild TBI (70–90%), approximately 10% to 30% suffer from moderate or severe TBI [5,6]. Regardless of the severity, individuals after TBI may suffer from short- or long-term impairments in cognition [7,8], psychosocial functioning [9], health-related quality of life (HRQoL) [10,11], mental health [12,13], and/or functional disability [14]. These impairments can be assessed using domain-specific outcome measures.

The data analyzed in this study were collected in the international Collaborative European NeuroTrauma Effectiveness Research in TBI observational study (CENTER-TBI; clinicaltrials.gov NCT02210221), which has been conducted since 2014 in 18 European countries and Israel, with enrolment being completed at the six-month outcome assessment in 2018. This study aimed to capture a contemporary picture of TBI with respect to all severity groups, its care and outcome, to develop precision medicine approaches and apply comparative effectiveness research to identify best practices. It provides insights into the longitudinal detection of somatic, functional, behavioral, psychiatric, cognitive, psychological, and psychosocial sequelae after TBI and can serve as a basis for the development of a new multidimensional assessment approach [15,16].

An important criterion when selecting instruments for research and clinical practice is their psychometric quality. For most patient-reported outcome measures (PROMs) administered in the CENTER-TBI study this had not yet been examined in the field of TBI, nor had the newly translated versions of the instruments been psychometrically investigated. Hence, the present study aims to investigate the classical psychometric properties of the newly and previously translated PROMs in the field of TBI administered in the CENTER-TBI study.

In research and clinical contexts, instruments offer insights into outcome after TBI. The comparability of the translated instruments with their original version and the validation in the field of TBI enables the reliable and valid aggregation of data in multi-center national and international studies on outcomes after TBI.

The study aims are the investigation of:

1. The *reliability* (total score, scale, and item level) of the PROMs, comparing them with the values of the original instrument versions to ascertain the quality and comparability of the translations and applicability in the field of TBI;
2. The *convergent* and *discriminant validity* of the PROMs with established measures assessing functional recovery after TBI (GOSE), generic HRQoL (SF-36v2/SF-12v2), and TBI severity (GCS);
3. The *factorial validity* using confirmatory factor analyses (CFA) to replicate the original factorial structure of the translated instruments.

## 2. Materials and Methods

### 2.1. Participants

Participants were recruited at 63 centers across 18 countries, from 19 December 2014 to 17 December 2017. Ethical approval was secured for each site and informed consent was obtained from all patients or from their legal representatives. The inclusion criteria for the core study were a clinical diagnosis of TBI, presentation within 24 h after injury, and an indication for a computed tomography (CT) scan. Patients were differentiated into three strata: emergency room (ER; patients primarily evaluated at an ER), admission (ADM; patients admitted primarily to a hospital ward), and intensive care unit (ICU; patients who were primarily admitted to an ICU). Further details can be found elsewhere [16]. Data were retrieved from the core 2.1 of the CENTER-TBI database using the data access tool Neurobot.

The core study sample included 4509 individuals. In the present study, we focused on participants aged 16 years and above who had completed at least one outcome measure at the six months’ assessment after the TBI. The data were collected either on-site at the hospital by personnel, by face-to-face or telephone interviews (clinical ratings), or via mail (PROMs) and centrally entered using a web-based electronic case report form.

### 2.2. Sample Charachteristics

Language, sex, age, education, employment, marital status, and living situation were selected as sociodemographic characteristics. Samples were then aggregated by language. More specifically, individuals from German-speaking communities in Austria, Belgium, and Germany were integrated into the German sample, individuals from French-speaking communities in Belgium and France into the French sample, and individuals from Dutch-speaking communities in Belgium or the Netherlands were merged into the Dutch sample. Only few participants (*N* = 20) received the outcome questionnaires in a language other than in the local language of the participating site. These individuals were classified according to their respective language group: Dutch (7), English (8), German (1), Romanian (3), and Swedish (1).

The following variables were used to characterize extracranial and brain injuries: the individuals’ mental health status before the injury, clinical care pathways, cause of injury, loss of consciousness (LOC), post-traumatic amnesia (PTA), TBI severity (GCS), abnormalities on computed tomography (CT) scans, total injury severity score (ISS), and brain injury severity score from the Abbreviated Injury Scale (AIS) [17].

### 2.3. Pataient-Reported Outcome Measures (PROMs)

Since most instruments applied in the CENTER-TBI study only existed in English, they had to be translated into the languages of the participating countries following a formalized approach (i.e., linguistic validation) to ensure their linguistic, cultural and conceptual comparability in the respective languages [18,19]. For more details, see von Steinbuechel et al. [20].

The selection of the outcome measures was informed by the Common Data Elements (CDE) recommendations [21,22]. For six out of eight PROMs (see instrument description marked with an asterisk * below), at least one translation had to be performed. In this study, we report psychometrics for all eight PROMs newly and previously translated yet not validated instruments in the field of TBI.

The Generalized Anxiety Disorder 7 Item Scale (GAD-7)* [23] measures the level of generalized anxiety disorder using seven items and a four-point Likert scale (from 0 “not at all” to 3 “nearly every day”). The total score ranges from 0 to 21 with values of 10 and above indicating impairment and cut-offs of 5, 10, and 15 representing mild, moderate, and moderately severe to severe anxiety, respectively [23].

The Patient Health Questionnaire (PHQ-9)* [24] assesses self-reported symptoms of major depression using nine items and a four-point Likert scale (from 0 “not at all” to 3 “nearly every day”). The PHQ-9 total score ranges from 0 to 27 with a score of 10 and above indicating clinically relevant impairment and cut-offs of 5, 10, 15, and 20 indicating mild, moderate, moderately severe, and severe depression, respectively [24,25].

Both the GAD-7 and PHQ-9 were available in almost all languages except for Latvian (GAD-7 and PHQ-9) and Serbian (GAD-7 only). Nevertheless, we conducted analyses on both instruments to examine their psychometric properties in individuals after TBI.

The Posttraumatic Stress Disorder Checklist-5 (PCL-5)* [26] comprises 20 symptoms of post-traumatic stress disorder (PTSD) based on the Diagnostic and Statistical Manual of Mental Disorders, 5th edition (DSM-5) [27], using a five-point Likert scale (from 0 “not at all” to 4 “extremely”). The total score ranges from 0 to 80 with higher values indicating greater impairment. For clinical screening, either a cut-off score of 31 [28] or 33 is applied [29].

The Rivermead Post-Concussion Symptoms Questionnaire (RPQ)* **[30]** uses a five-point Likert scale (from 0 “not experienced at all” to 4 “a severe problem”) to evaluate the following 16 post-concussion symptoms: headaches, dizziness, nausea and/or vomiting, noise sensitivity, sleep disturbance, fatigue, irritability, depression, frustration, forgetfulness and poor memory, poor concentration, slow thinking, blurred vision, light sensitivity, double vision, and restlessness. Participants rate how much they have been suffering from these symptoms during the past 24 h compared with their condition before the accident. The RPQ total score ranges from 0 to 64 with cut-offs of 13, 25, and 33 indicating mild, moderate, and severe symptoms, respectively [31].

The Quality of Life after Brain Injury Scale (QOLIBRI)* [32,33] measures TBI-specific HRQoL in individuals after TBI. It consists of six domains comprising 37 items using a five-point Likert scale (from 0 “not at all” to 4 “very”). The six domains comprise cognition, self, daily life and autonomy, social relationships, emotions, and physical conditions. The total score is transformed linearly to range from 0–100, whereby higher values indicate better TBI-specific HRQoL [34]. Patients after TBI with a score below 60 may be assumed to display impaired HRQoL [34]; country-specific reference values can be found elsewhere [35]. For the QOLIBRI, psychometric criteria of almost all target language versions involved in the present study (except for Swedish) had already been published [32,36]. The Spanish translation was published after CENTER-TBI had started [37]. To be congruent with the analyses of other PROMs, we replicated the psychometric analyses for the nine language versions of the QOLIBRI.

The Quality of Life after Brain Injury—Overall Scale (QOLIBRI-OS)* [38] is the short version of the QOLIBRI measuring the physical condition, cognition, emotions, daily life and autonomy, social relationships, and current and future prospects with using six items. The items are answered on a five-point Likert scale (from 0 “not at all” to 4 “very”). Patients after TBI with a score below 52 may be assumed to display impaired HRQoL [34]; country-specific reference values can be found elsewhere [39]. For the QOLIBRI-OS too, psychometric properties have already been examined in almost all languages, except for Spanish and Swedish [38]. Here, again, psychometric analyses were replicated in all languages to be congruent with the other PROMs.

The 36-item Short Form Health Survey—Version 2 (SF-36v2) [40,41]. The SF-36v2 measures subjective health status using 36 items with various response formats for each of the eight scales (from dichotomous “yes/no” to polytomous five-point Likert scale responses). The scales can be summed to produce the physical component score (PCS) and mental component score (MCS) measuring physical and mental functioning, respectively. Both scores range from 0 to 100 with higher values indicating better HRQoL. The values can be transformed into *T*-scores (*M* = 50, *SD* = 10) based on a normative U.S. sample. A value below 47 on a single health domain scale or component summary score is indicative of functional impairment in comparison to the U.S. population [40].

The 12-Item Short Form Survey—Version 2 (SF-12v2) [42] is a short, 12-item version of the SF-36v2. The scores range from 0 to 100 with higher values indicating better HRQoL. The raw values can be transformed into *T*-scores (*M* = 50, *SD* = 10) based on a normative U.S. sample. However, the authors recommend using country- and group-specific cut-off values as not every country/group has a mean health of 50 [42,43]. In the CENTER-TBI study, the SF-12v2 was found to have more missing data than the SF-36v2. Therefore, to increase the power for the calculation of the PCS and MCS of the SF-12v2, missing values were replaced by values derived from the respective items of the SF-36v2 and combined with reported data. For the analyses on the item level, only reported data were used.

The SF-36v2 and SF-12v2 translations were already available in the target languages and had to be purchased from Optum for one-time use [44]. However, since most translated versions of both the SF-36v2 and the SF-12v2 were not subjected to psychometric analyses in the field of TBI, they were included in the analyses of the present study. Both instruments were also used for validity analyses.

### 2.4. Clinician-Reported Outcome (ClinRo) and a Clinical Scale

The instruments listed below were used to analyze convergent and discriminant validity.

The Glasgow Outcome Scale Extended (GOSE) [45] is a clinician-reported outcome (ClinRo) of functional recovery after TBI using an eight-point scale (1 = dead, 2 = vegetative state, 3/4 = lower/upper severe disability, 5/6 = lower/upper moderate disability, 7/8 = lower/upper good recovery) and is based on structured interviews (GOSE) or self-ratings by individuals after TBI or their proxy (the questionnaire version; GOSE-Q [46]). Missing GOSE values were centrally replaced by values derived from the GOSE-Q. Since the GOSE-Q is not able to differentiate between vegetative state and lower severe disability, GOSE levels 2 and 3 were collapsed into one category. The missing values at six-months outcome assessments were imputed using a multi-state model; the imputation procedure is described elsewhere [47]. The GOSE was not subjected to reliability analyses, as it would require data from independent raters to provide interrater reliability, which was not available in the CENTER-TBI database.

The Glasgow Coma Scale (GCS) [48] allows healthcare professionals to consistently evaluate the level of consciousness of individuals after TBI, also classifying the severity of TBI. The GCS scores range from 3 (no response) to 15 (normal level) with higher values indicating less impaired consciousness and lower TBI severity. Scores of 13 to 15 indicate mild TBI, 9 to 12 moderate TBI, and 3 to 8 severe TBI.

### 2.5. Statistical Analyses

**Figure 1:**
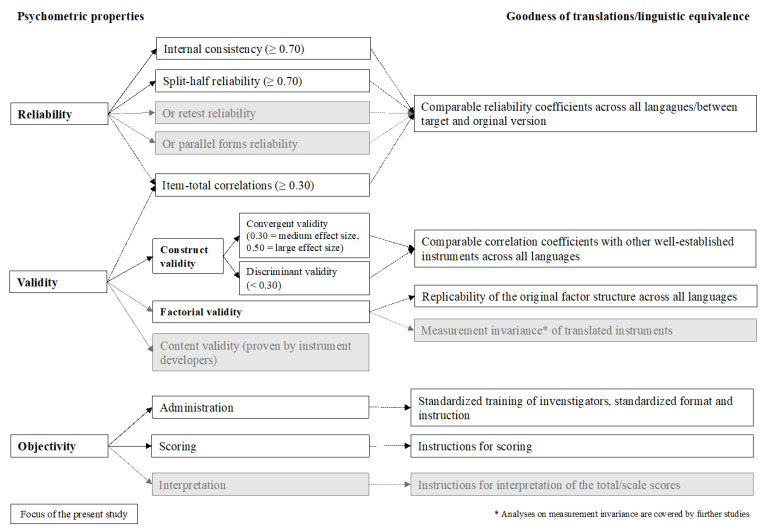
Criteria of classical test theoretical psychometric analyses and their application in this study. The white boxes indicate analyses performed in this study; the grey boxes describe psychometric properties investigated either during instrument development (i.e., content validity), or alternative methods of retest reliability or parallel form reliability, or analyses deferred to further studies (i.e., measurement invariance and interpretation).

The present study focuses on the analyses of reliability, convergent and discriminant validity of eight PROMs in nine TBI language samples with enough participants (i.e., at least 50 participants in the Dutch, English, Finnish, French, German, Italian, Norwegian, Spanish, and Swedish samples) as well as factorial validity in six samples (i.e., at least 150 participants in the Dutch, English, Finnish, Italian, Norwegian, and Spanish samples). Figure 1 provides an overview of our psychometric analyses according to the classical test theoretical (CTT) criteria with the respective cut-off values [49].

### 2.6. Descriptive Statistics

Descriptive statistics include information on the sample sizes, percentage of missing data, mean (*M*), standard deviation (*SD*), skewness (*SK*), and kurtosis (*KU*) for each item per language version of an instrument and an average of the item characteristics across all languages. For skewness, values less than −1 or greater than 1 indicate a highly skewed distribution; values from ±1 to ±0.5 show that the distribution is moderately skewed; values from −0.5 to +0.5 denote a symmetrical distribution. For asymmetry and kurtosis, values between −2 and +2 are considered acceptable [50].

### 2.7. Reliability

For reliability analyses, researchers often accept data of 30 participants as being sufficient to detect a required minimal effect of 0.70 as a cut-off value for reliability coefficients [51]. However, some researchers argue that larger sample sizes are required to avoid bias [51,52]. In the present study, reliability coefficients were therefore only calculated if the sample size comprised at least 50 individuals per language, to provide more robust results.

To examine the reliability of each instrument, Cronbach’s alpha, split-half reliability with the Spearman–Brown correction (odd vs. even items), and Cronbach’s alpha if an item is omitted were reported. Both, the split-half reliability and the Cronbach’s alpha if item omitted were calculated for scales with at least three items. Although different recommendations in terms of cut-off points for the Cronbach’s alpha do exist, there is an agreement that in group comparisons Cronbach’s alpha should reach at least a value of 0.70 implying acceptable internal consistency [53]; an alpha above 0.90 indicates excellent internal consistency [54]. The Cronbach’s alpha value, if an item has been omitted, should not exceed the total Cronbach’s alpha of a scale. A value higher than the total Cronbach’s alpha indicates that the excluded item decreases the reliability of the instrument and requires further revision [55].

To evaluate the discriminating ability of the items, item–total correlations either at the scale or at the total score level, or both were calculated. A correlation coefficient of 0.30, corresponding to a medium effect size, was chosen as the cut-off criterion, based on the guidelines for effect size proposed by Cohen [56,57]. An item–total correlation below 0.30 implies that the item cannot discriminate well between high-performing and low-performing individuals. Furthermore, low item–total correlations, especially at the scale level, may identify irregularities of the factorial structure of an instrument.

### 2.8. Validity

#### 2.8.1. Convergent and Discriminant Validity

All language samples analyzed in this study included at least 50 observations, which is recommended for validity analyses [58].

Spearman correlation coefficients were used to examine associations between the GOSE, physical (PCS) and the mental component score (MCS) of the SF-36v2 and SF-12v2, and the total scores/domain-specific scores of all other measures.

Discriminant validity was investigated by calculating Spearman correlation coefficients for the GCS and the total and scale scores of all instruments, to be in line with analyses already provided in the field of TBI [59]. To evaluate the strength of correlations, the Cohen criteria [56,57] were applied to identify small (0.10), medium (0.30), and large (0.50) effect sizes.

#### 2.8.2. Factorial Validity

Factorial validity was examined by means of confirmatory factor analyses (CFA) and a robust weighted least squares estimator (WLSME) for ordinal data, whereby only the original factor structure of the instruments was analyzed. Therefore, one-factor solutions were estimated for the GAD-7 [23], the PHQ-9 [24], the RPQ [30], and the QOLIBRI-OS [38]. For the other instruments, respective multiple scale models were inspected: a four-factor model for the PCL-5 [26], a five-factor model for the QOLIBRI [32], an eight-factor model with two second-order factors for the SF-36v2 [40], and finally, a two-factor model for the SF-12v2 [42]. In CFA analyses, samples should comprise at least 150 observations to provide stable results [60]. Therefore, only language samples fulfilling this criterion were analyzed.

The model fit was evaluated based on the following fit indices using the respective cut-off values (in paratheses): χ^2^ statistics with respective *p*-values (*p* > 0.01) [61], comparative fit index (CFI > 0.95) [62], Tucker–Lewis index (TLI > 0.95 [63]) root mean square error of approximation (RMSEA < 0.06) [64] with a 90-percent confidence interval (CI), and standardized root mean square residual (SRMR < 0.08) [63]. As some of the fit indices may be biased (e.g., χ^2^ test can be influenced by large sample size [61]), all indices were considered simultaneously to evaluate the model fit. Furthermore, item loadings over 0.50 were considered acceptable and over 0.70 desirable [53].

Analyses were performed using the packages psych [65] for psychometric characteristics and lavaan [66] for the factorial validity analyses applying the R version 4.0.2 [67].

### 2.9. Comparability of the Translated Versions

To evaluate the quality of the translated versions of the eight PROMs, psychometric criteria obtained from the CENTER-TBI language samples were compared with those reported for the original English instrument versions. For this purpose, a systematic literature search was carried out. Psychometric characteristics were compared with those obtained from the original validation studies in the original populations, for which the respective instrument was developed. If available, they were also compared with the validation studies in the field of TBI. If the original articles did not provide information on all coefficients, these were retrieved from more recent studies.

These comparisons were confined to the reliability coefficients (i.e., Cronbach’s alpha coefficients, split-half or test–retest reliability), as validity testing in the original studies was performed using instruments not applied in the CENTER-TBI study. Instruments showing reliability within the same ranges (i.e., <0.70—acceptable, 0.70–0.89—good, ≥0.90—excellent) or higher in both original and the PROMs applied in the CENTER-TBI study were considered comparable.

## 3. Results

### 3.1. Sample Characteristics

**Figure 2:**
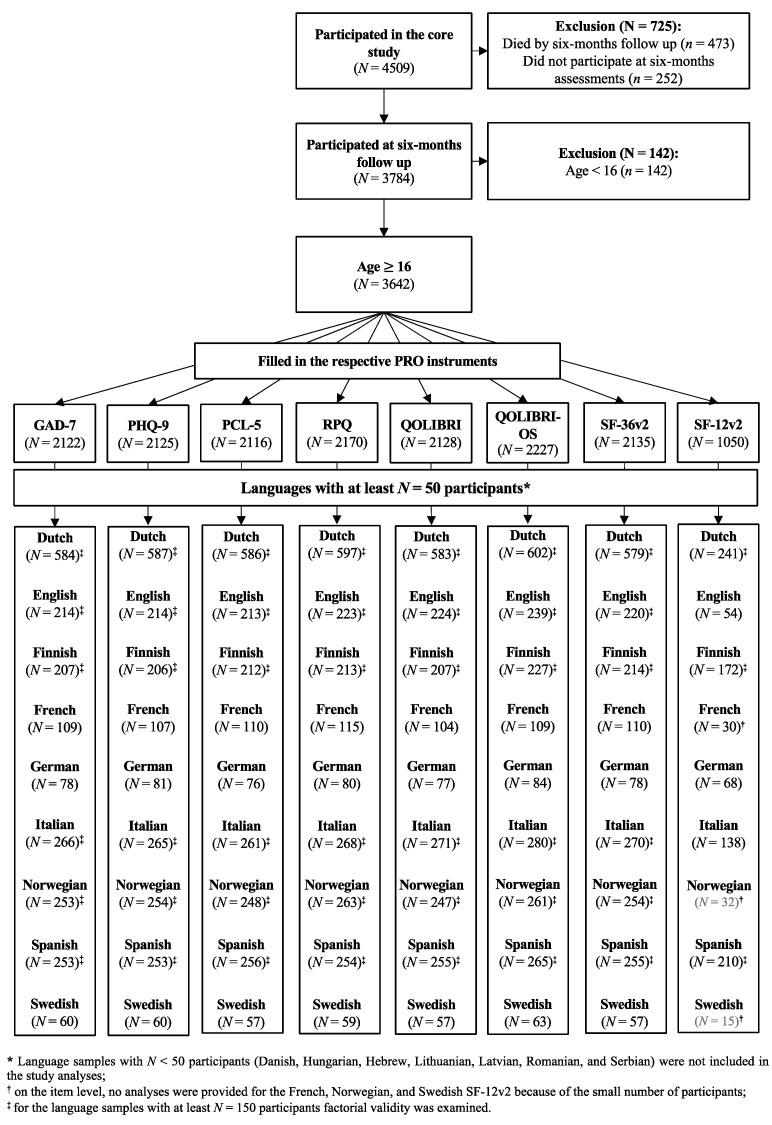
Number of participants for each PROM and language.

For the CENTER-TBI study, eight PROMs were translated or already available in 20 target languages (Figure 2). As some countries withdrew from the project early (Bulgarian and Czech centers) or no participants were recruited (Arabic and Russian), 16 countries participated in the study. Seven out of 16 language samples (i.e., Danish, Hungarian, Hebrew, Lithuanian, Latvian, Romanian, and Serbian) were not psychometrically analyzed due to a low number of observations (*N* < 50). Additionally, three language samples (French, Norwegian, Swedish) had to be excluded from the reliability analyses of the SF-12v2, also because of insufficient sample sizes. For the factorial validity, six language samples comprising at least *N* = 150 observations (i.e., Dutch, English, Finnish, Italian, Norwegian, and Spanish) were investigated for all instruments except for the SF-12v2, as only three SF-12v2 language samples (i.e., Dutch, Finnish, and Spanish) fulfilled the sample size criteria.

The number of participants varied between PROMs, since not every participant filled in each instrument at the six-months outcome assessment. Sample characteristics for each instrument and language are provided in the Online Supplement (OS 1: Sample characteristics, Tables S1–S8). A brief overview on the sample compositions used for the analyses is presented in Figure 2. Appendix A (Table A1) provides additional information on the number of participants for the validity analyses using the GOSE and the GCS.

### 3.2. Reliability and Comparability of the PROMs

Reliability coefficients for the total and scale scores of the PROMs are shown below. Item characteristics as well as reliability coefficients on the item level are reported in the respective tables in the Online Supplement 2 (OS-2 Reliability, Tables S1–S8).

#### 3.2.1. GAD-7

All translations analyzed were available prior to the CENTER-TBI study. Item scores for the GAD-7 were not normally distributed (*SK*: *M* = 1.64, *SD* = 0.51; *KU*: *M* = 2.40, *SD* = 2.22) across all languages. At the item level, most items were moderately to strongly correlated with the total score of the GAD-7 in most languages (0.36 to 0.89). When calculating Cronbach’s alpha if item omitted, all values were smaller than the total Cronbach’s alpha across all languages. The values of the split-half reliability ranged from 0.70 to 0.90 across all languages. On the total score level, all translations revealed Cronbach’s alpha and split-half reliability values comparable to the results of the original English versions in a non-TBI population (i.e., patients from 15 primary care sites [23]) except for the Finnish, German, Spanish, and Swedish versions showing Cronbach’s alpha values slightly lower than 0.90, but over 0.80. The reliability results were within the same or higher range (0.70 to 0.89 and ≥0.90) compared to the validation in an English TBI sample [68] (see Table 1).

#### 3.2.2. PHQ-9

All analyzed PHQ-9 translations were available prior to the CENTER-TBI study. The items of the PHQ-9 were not normally distributed (*SK*: *M* = 1.66, *SD* = 0.80; *KU*: *M* = 2.71, *SD* = 3.85) across all languages. At the item level, all items were moderately to highly correlated with the total scores of the PHQ-9 across all languages, except for Swedish. Here, the item “*Moving or speaking so slowly that other people could have noticed”* had a low correlation (*r* = 0.18) with the total score. At the total score level, the Cronbach’s alpha values were above 0.70 (0.78 to 0.89) in every language. When calculating Cronbach’s alpha if item omitted, no value exceeded the total Cronbach’s alpha. The values of the split-half reliability ranged from 0.85 to 0.90. Reliability coefficients were comparable (i.e., ranged from 0.70 to 0.89 and above) with those obtained from the original English publication in a non-TBI population (i.e., primary care patients from five general health clinics and three family practice clinics) [24]. The Cronbach’s alpha coefficients calculated from CENTER-TBI data were slightly lower compared with the results from the first English validation study in a TBI sample [69], whereas the results of the split-half reliability were within a comparable range [70] (see Table 2).

#### 3.2.3. PCL-5

All but the Norwegian version of the PCL-5 were translated for the CENTER-TBI study. The items of the PCL-5 were not normally distributed (*SK*: *M* = 1.75, *SD* = 0.66; *KU*: *M* = 2.70, *SD* = 2.99) across all languages. At the scale (i.e., DSM-5 cluster) level, most items had medium to high correlations with the cluster total scores of the PCL-5 across all languages. Only the item “*Trouble remembering important parts of the stressful experience*” displayed borderline correlations with the total cluster scores in French (r = 0.20), Norwegian (r = 0.28), and Swedish (r = 0.28) language samples. The internal consistency was satisfactory to excellent (0.74 to 0.92) at the cluster level. All split-half reliability coefficients demonstrated at least satisfactory reliability (i.e., ≥0.70). At the total score level, the values of the Cronbach’s alphas ranged from 0.91 to 0.94 in all languages. The Cronbach’s alphas if item omitted did not exceed the values of the initial Cronbach’s alpha except for the item “*Trouble remembering important parts of the stressful experience”* in all but English and German language samples. The split-half reliability was excellent (0.92 to 0.96) across all languages. Cronbach’s alpha coefficients on the total score and the cluster level were comparable to the original English validation results in a non-TBI sample (i.e., undergraduate students having experienced a stressful life event [26] and military service members [71]) in all translations. No publications on psychometric properties of the PCL-5 in the field of TBI samples were found (see Table 3).

#### 3.2.4. RPQ

All but the German and Norwegian versions of the RPQ were translated for the CENTER-TBI study. The item score distributions of the RPQ were skewed (*SK*: *M* = 1.31, *SD* = 0.83; *KU*: *M* = 1.37, *SD* = 4.07) across all languages. At the item level, most items displayed medium to high correlations with the total scores of the RPQ. In the German translation, the item “*Double Vision”* had a borderline correlation with the total score of the RPQ (r = 0.25). The item “*Nausea”* of the German and Swedish translations displayed rather low correlations (r = 0.25 and r = 0.24, respectively) with the total score.

At the scale level, however, the values of the Cronbach’s alpha and the split-half reliability were above 0.70 across all languages. No comparisons between the original and the translated language versions can be provided for the internal consistency, as no information was available concerning Cronbach’s alpha in the English RPQ version investigated in a TBI sample. Moreover, further studies on the RPQ [31,72,73] provided no information on the internal consistency, as they focused on the factorial structure of the questionnaire. The test–retest reliability scores in the original study were comparable to the split-half reliability results of the English and Finnish language samples from the CENTER-TBI study. The split-half reliability of all other translations was slightly above 0.90 except for the Swedish version ($\alpha_{\mathrm{Cronbach}}$ = 0.82). For details, see Table 4.

#### 3.2.5. QOLIBRI

At the total score level, Cronbach’s alpha and the split-half reliability coefficients of all translated QOLIBRI versions were above 0.90. Item–total correlations displayed medium to high correlations with the total score except for the German version. Here, the item “*How bothered are you by feeling angry or aggressive*” revealed a low correlation with the total score (r = 0.25). Below, item distributions and reliabilities are reported for each subscale.

*Cognition*. The items were almost normally distributed (*SK*: *M* = −0.91, *SD* = 0.34; *KU*: *M* = 0.46, *SD* = 0.78) across all languages and highly correlated with the total score of the Cognition scale across all languages (0.62 to 0.84). At the scale level, all reliability coefficients were excellent (Cronbach’s alpha: 0.91 to 0.93; split-half-reliability: 0.90 to 0.94).

*Self*. The items were approximately normally distributed (*SK*: *M* = −0.68, *SD* = 0.27; *KU*: *M* = −0.11, *SD* = 0.65) in all languages and correlated highly with the scale score (0.64 to 0.88). Reliability coefficients were excellent (Cronbach’s alpha: 0.92 to 0.94; split-half reliability: 0.92 to 0.96).

*Daily Life and Autonomy*. Across all languages, items were nearly normally distributed (*SK*: *M* = −0.97, *SD* = 0.36; *KU*: *M* = 0.18, *SD* = 0.91). Items correlated highly with the scale scores (0.61 to 0.86). Reliability coefficients were excellent (Cronbach’s alpha: 0.90 to 0.94; split-half reliability: 0.92 to 0.96).

*Social Relationships*. In general, item scores were normally distributed (*SK*: *M* = −1.02, *SD* = 0.40; *KU*: *M* = 0.61, *SD* = 1.25) across all languages. Correlations for item and scale scores ranged from 0.39 to 0.80. Reliability results were satisfactory to excellent for all translated versions (Cronbach’s alpha: 0.76 to 0.89; split-half reliability: 0.86 to 0.95).

*Emotions*. On average, the items were nearly normally distributed (*SK*: *M* = −1.01, *SD* = 0.48; *KU*: *M* = 0.25, *SD* = 1.20) for all languages. All items were moderately to highly correlated with the total scores of the scale across all languages (0.52 to 0.82). At the scale level, reliability results were good to excellent (Cronbach’s alpha: 0.82 to 0.89; split-half reliability: 0.86 to 0.90).

*Physical*. The item distributions were close to a normal distribution (*SK*: *M* = −0.89, *SD* = 0.38; *KU*: *M* = −0.23, *SD* = 0.88) across all languages. All coefficients were satisfactory to good across all languages on the item (item–total correlation: 0.35 to 0.74) as well as on the scale level (Cronbach’s alpha: 0.76 to 0.88; split-half reliability: 0.76 to 0.88).

All reliability coefficients were comparable (i.e., within the same or higher range) to those reported in the original publication on a TBI population. As the QOLIBRI was developed for use in the TBI field, no validation studies in non-TBI populations are reported (see Table 5).

#### 3.2.6. QOLIBRI-OS

The items of the QOLIBRI-OS were close to being normally distributed (*SK*: *M* = −0.71, *SD* = 0.23; *KU*: *M* = −0.05, *SD* = 0.53) and were moderately to highly correlated with the total scores of the QOLIBRI-OS (0.59 to 0.83) across all languages. At the total score level, the Cronbach’ alpha values were close to or above 0.90 (0.88 to 0.92), and the split-half reliability ranged from 0.90 to 0.94. Moreover, the values of the Cronbach’s alpha if item omitted were smaller than the Cronbach’s alpha in each language. The reliabilities of the translated versions were in general within the same range as those of the original ones. The split-half coefficients were greater than the test–retest reliability of the original QOLIBRI-OS. For details, see Table 6.

#### 3.2.7. SF-36v2

All SF-36v2 translations were available prior to the CENTER-TBI study. The instrument was investigated on the scale and item level and with respect to the mental (*MCS*) and physical (*PCS*) component score.

*Physical Functioning (PF).* The items were not normally distributed (*SK*: *M* = −1.46, *SD* = 0.88; *KU*: *M* = 1.65, *SD* = 3.27) across all languages. Items were moderately to highly correlated with the scale score across all languages (0.56 to 0.91). At the scale level, all reliability coefficients showed excellent results (Cronbach’s alpha: 0.92 to 0.95; split-half-reliability: 0.95 to 0.98).

*Role-Physical (RP).* The items were almost normally distributed (*SK*: *M* = -0.50, *SD* = 0.35; *KU*: *M* = −0.90, *SD* = 0.45) across all languages and highly correlated with the scale score across all languages (0.83 to 0.93). At the scale level, all reliability coefficients were excellent (Cronbach’s alpha: 0.94 to 0.96; split-half-reliability: 0.94 to 0.97).

*Bodily Pain (BP).* The items were almost normally distributed (*SK*: *M* = −0.65, *SD* = 0.33; *KU*: *M* = −0.60, *SD* = 0.59) across all languages and highly correlated with the scale score across all languages (0.78 to 0.83). At the scale level, all reliability coefficients showed were good to excellent (Cronbach’s alpha: 0.86 to 0.89). The split-half reliability was not calculated because of the scale length (two items).

*General Health (GH).* The items were normally distributed (*SK*: *M* = −0.60, *SD* = 0.53; *KU*: *M* = −0.31, *SD* = 0.88) across all languages. Items were moderately to highly correlated with the scale score across all languages (0.37 to 0.78). At the scale level, Cronbach’s alpha was satisfactory to good (0.73 to 0.84). The split-half reliability of the English, German, Norwegian, Spanish, and Swedish samples was low to borderline (0.59 to 0.69) and satisfactory for the other languages (0.70 to 0.78).

*Vitality (VT).* The items were normally distributed (*SK*: *M* = −0.28, *SD* = 0.25; *KU*: *M* = −0.50, *SD* = 0.37) across all languages and moderately to highly correlated with the scale score across all languages (0.48 to 0.78). At the scale level, all reliability coefficients were good to excellent (Cronbach’s alpha: 0.83 to 0.88; split-half reliability: 0.85 to 0.95).

*Social Functioning (SF).* The items were normally distributed (SK: *M* = −0.89, *SD* = 0.34; *KU*: *M* = −0.12, *SD* = 0.83) across all languages. The items were highly correlated with the scale score across all languages (0.69 to 0.81). Cronbach’s alpha was good to excellent (0.81 to 0.90); split-half reliability was not calculated because of the scale length (two items).

*Role Emotional (RE).* Across all languages, items were nearly normally distributed (*SK*: *M* = −1.00, *SD* = 0.40; *KU*: *M* = 0.09, *SD* = 0.91) in all language samples. They were highly correlated with the scale score across all languages (0.72 to 0.92). At the scale level, all reliability coefficients were excellent (Cronbach’s alpha: 0.90 to 0.95; split-half reliability: 0.91 to 0.96).

*Mental Health (MH).* The items were close to being normally distributed (*SK*: *M* = −0.80, *SD* = 0.45; *KU*: *M* = 0.22, *SD* = 1.11) across all languages. They were moderately to highly correlated with the scale score across all languages (0.48 to 0.83). At the scale level, all reliability coefficients were good to excellent (Cronbach’s alpha: 0.83 to 0.89; split-half reliability: 0.81 to 0.92).

The internal consistency of the translated versions of the SF-36v2 was comparable to the original English version, which was validated in a U.S. general population [40,41]. The Cronbach’s alpha coefficients on the scale levels were within the same ranges or above. The split-half reliability coefficients were within the same or higher ranges compared to the original version. Despite the wide application of the SF-36v2, no studies on psychometric properties of the English version in the field of TBI for the English version were found.

*Physical Component Score (PCS).* Items were moderately to highly correlated with the PCS (0.35 to 0.87) except for the item “*I expect my health to get worse*” in the English version (r = 0.23). Cronbach’s alpha ranged from 0.32 to 0.95 and the split-half reliability coefficients from 0.93 to 0.95. When omitting an item, the newly calculated Cronbach’s alpha did not exceed the initial value in any language sample. The reliability coefficients were within the same or higher range compared with the psychometric properties of the original SF-36v2.

*Mental Component Score (MCS).* The items were moderately to highly correlated with the MCS (0.43 to 0.88). Cronbach’s alpha (0.92 to 0.95) and split-half coefficients (0.95 to 0.98) indicted a high reliability. When omitting an item, the newly calculated Cronbach’s alpha values did not exceed the initial one. Here, again, the reliability of the instrument translations was comparable (i.e., was within the same or higher range) with the results obtained from the original validation study (see Table 7).

#### 3.2.8. SF-12v2

All SF-12v2 translations were available prior to the CENTER-TBI study. Many of the scales of the SF-12v2 consist of two items (PF, RP, RE, MH), and some include one item (BP, VT, SF, GH); therefore, the reliability coefficients are provided on the physical (PCS) and mental (MCS) component score level.

*Physical Component Score (PCS)*. The items were close to being normally distributed (*SK*: *M* = −0.54, *SD* = 0.47; *KU*: *M* = −0.64, *SD* = 0.55) across all languages. On the item level, all items correlated moderately to highly with the PCS (0.55 to 0.89). At the scale level, all reliability coefficients were good to excellent (Cronbach’s alpha: 0.86 to 0.94; split-half-reliability: 0.88 to 0.92).

*Mental Component Score (MCS)*. The items were close to being normally distributed (*SK*: *M* = −0.67, *SD* = 0.35; *KU*: *M* = −0.32, *SD* = 0.49) across all languages and correlated moderately to highly with the MCS (0.55 to 0.89). At the scale level, all reliability coefficients were good to excellent (Cronbach’s alpha: 0.86 to 0.94; split-half-reliability: 0.88 to 0.92).

The reliability of the translated versions of the SF-12v2 was comparable to the original English version, which was validated in a general U.S. population [42]. The split-half reliability coefficients (using the CENTER-TBI data) were within the higher range for both component scores compared with the original version. Despite the wide application of the SF-12v2, no studies on psychometric properties of the English version in the field of TBI were found (see Table 8).

### 3.3. Validity

#### 3.3.1. Convergent and Discriminant Validity

Validity coefficients for all PROMs and the PCS and MCS of the SF-36v2 and the SF-12v2 are provided on the total score level (see Table 9). For details concerning the validity of the PCL-5, the QOLIBRI, and the SF-36v2 on the scale level, see Appendix B Table A2, Table A3 and Table A4.

Most instruments indicating a degree of impairment (i.e., GAD-7, PHQ-9, PCL-5, and RPQ) displayed medium to high negative correlations with the PCS of the SF-36v2 (−0.30 to −0.82). Some exceptions were observed in the English (r_S_ = −0.15) and the Swedish (r_S_ = −0.12) versions of the GAD-7, as well as in the French version (r_S_ = −0.25) of the PCL-5 which demonstrated low negative correlations. For the instruments measuring disease-specific HRQoL after TBI (i.e., the QOLIBRI and the QOLIBRI-OS) medium to high positive correlations with the SF-36v2 PCS domain (0.49 to 0.65) were found across all languages.

All PROMs indicating a degree of impairment correlated negatively and moderately to highly with the MCS of the SF-36v2 (−0.44 to −0.83). The ones capturing disease specific HRQoL displayed medium to high positive correlations (0.57 to 0.80) with the MCS across all languages.

The PCS of the SF-12v2 was negatively correlated at a low to medium level with the PHQ-9 (−0.32 to −0.56) and RPQ (−0.40 to −0.55) and positively with the QOLIBRI (0.49 to 0.65) and the QOLIBRI-OS (0.48 to 0.61) across all languages. The GAD-7 revealed significant medium correlations with the PCS of the SF-12v2 in Finnish (r_S_ = −0.34), in German (r_S_ = −0.31), and in Spanish (r_S_ = −0.40); all other values ranged from −0.29 to −0.15.

All PROMs indicating a degree of impairment were negatively and moderately to highly correlated with the MCS of the SF-12v2 (−0.42 to −0.79) and positively with the QOLIBRI and the QOLIBRI-OS (0.58 to 0.74).

Significant medium to high correlations were found between the PROMs and the GOSE total score, whereby greater impairment was associated with lower functional recovery status in almost all languages across all instruments (from −0.30 to −0.63). Only the German version of the GAD-7 (r_S_ = −0.24) and the German version of the PCL-5 (r_S_ = −0.16) demonstrated low associations with the GOSE. Higher TBI-specific HRQoL was associated with a better functional recovery status across all languages (0.37 to 0.64).

The associations of the PROMs and the GCS were weak and not significant in most languages. Only the Swedish translations of the GAD-7 (r_S_ = −0.30), the PHQ-9 (r_S_ = −0.33), the RPQ (r_S_ = −0.34), the QOLIBRI (r_S_ = 0.34), and the QOLIBRI-OS (r_S_ = 0.40) displayed medium correlations with the GCS.

#### 3.3.2. Factorial Validity

Table 10 gives an overview on the goodness of fit statistics for the estimated models. Factor loadings are provided in the Online Supplement (OS-3 Factorial validity, Tables S1–S8).

*GAD-7.* Except for the χ^2^ statistic and the RMSEA in the Dutch (χ^2^ only), English, Italian, and Spanish samples, the fit indices demonstrated that the data fitted the one-factor model well across the languages. The item loadings were above 0.50 (0.68 to 0.96) indicating that all items measured a unidimensional construct across the languages.

*PHQ-9.* Almost all indices exhibited a satisfactory model fit across the languages except for the χ^2^ statistic in the Dutch and Finnish translations and RMSEA and SRMR in the Finnish translation. The item loadings were above 0.50 (0.58 to 0.94) across all languages. Overall, the one-factor solution was acceptable.

*PCL-5.* Almost all fit measures exhibited a satisfactory model fit. The χ^2^ test of all translations was significant and the RMSEA of the Spanish translation was above the cut-off value. The model for the Finnish sample did not converge. Average item loadings on the scale (DSM-cluster) level were above 0.70 (B—*Intrusion*: 0.84 to 0.91; C—*Avoidance*: 0.88 to 0.91; D—*Negative alterations*: 0.75 to 0.81; E—*Hyperarousal*: 0.73 to 0.79) denoting an appropriate fit of the four-factor structure of the PCL-5 across all countries. However, the loadings of the item “*Trouble remembering important parts*” in the English (0.49) and Norwegian (0.38) translations were below the cut-off of 0.50.

*RPQ.* All RPQ translations revealed significant χ^2^ statistics and the RMSEA and SRMR values (except for the Dutch and Norwegian versions) were above the respective cut-offs. The factor loadings varied from 0.41 to 0.92. The item “*Headaches*” of the Finnish RPQ and the item “*Double Vision*” of the Norwegian RPQ reached values below the cut-off. Overall, the one-factor solution demonstrated a rather poor fit.

*QOLIBRI.* All but two (the English and Finnish) QOLIBRI translations had satisfactory fit indices, except for the χ^2^ statistic, which was significant across all translations. The English and Finnish models did not converge. The item loadings of the scales were above 0.70 (*Cognition*: 0.74 to 0.92; Self: 0.75 to 0.93; *Daily Life and Autonomy*: 0.76 to 0.96; Social: 0.65 to 0.92; *Emotions*: 0.63 to 0.97; *Physical*: 0.59 to 0.92). Overall, the original five-factor structure fitted the data well.

*QOLIBRI-OS.* For the most part, the CFA results of the QOLIBRI-OS translations displayed acceptable fit indices, with the RMSEA values of the Dutch, Italian, and Spanish translations slightly above the cut-off value and significant χ^2^ statistics. All other indices were within acceptable ranges. The factor loadings ranged from 0.73 to 0.92 indicating the unidimensionality of the TBI-specific HRQoL construct across the QOLIBRI-OS translations.

*SF-36v2.* Two out of six models did not converge (Finnish and Italian). The CFI and the TLI of the other translations were satisfactory; nevertheless, χ^2^ statistics were significant, and the RMSEA and the SRMR (except for the Dutch translation) were above the respective cut-off values. All factor loadings on the scale level were above 0.50; one item of the Dutch version of the SF-36v2 (“*Walking several hundred yards*”) was exceedingly highly correlated with the *Physical Functioning* scale and therefore also with the PCS (r = 1.0). Overall, the factorial structure of the SF-36v2 with eight scales and two second-order factors did not show evidence of a good fit.

*SF-12v2.* The models displayed satisfactory CFI and TLI values across all languages as well as the SRMR of the Spanish translation. The χ^2^ statistics were significant and the RMSEA and SRMR were above permissible cut-off values. The item loadings of the PCS ranged from 0.69 to 0.97 and of the MCS from 0.67 to 0.95.

## 4. Discussion

The present study examined psychometric properties of the eight PROMs administered in the CENTER-TBI study in individuals after TBI. Many of them were translated and linguistically validated for this study; others had not yet been psychometrically investigated in the field of TBI. Therefore, a classical test theorical framework was applied.

The results of the reliability and validity analyses performed on the PROMs indicate that most newly translated and already existing questionnaires generally displayed satisfactory to excellent psychometric characteristics in the field of TBI and were comparable to each other as well as to the original English versions investigated predominantly in non-TBI samples, in individuals after TBI, or both. On the scale level, high internal consistency and scale reliability of the newly translated and already existing instruments across all languages were observed. On the item level, only very few items from a few questionnaires demonstrated irregularities, mostly in no more than one language. However, the factorial validity analyses of the original instruments revealed some difficulties in the replicating the original factorial structures, indicating a need for further investigations.

Some translations displayed problems at the item level, displaying lower correlations with the respective total scale scores: the item “*Moving or speaking so slowly that other people could have noticed”* from the Swedish PHQ-9, the items “*Nausea”* in the Swedish and the German RPQ and “*Double Vision”* in the German RPQ, and the item “*How bothered are you by feeling angry or aggressive”* from the German QOLIBRI. Item–total correlations are directly related to the factorial structure of a questionnaire; therefore, low correlations may indicate that the questionnaire does not measure unidimensionally. The QOLIBRI consists of five scales; thus, the low correlation of the item “*How bothered are you by feeling angry or aggressive”* in the German translation is not problematic, as the scale level and total score level characteristics were satisfactory. Moreover, the low item–total correlations of the RPQ translations are not unexpected, as the questionnaire underwent several revisions regarding the scoring by different authors [30,72,73], whereby the items “*Nausea”* and “*Double Vision”* were assigned to different domains. Nevertheless, the low correlation of the item “*Moving or speaking so slowly that other people could have noticed”* in the Swedish PHQ-9 is more difficult to explain, as the PHQ-9 is a unidimensional measure. Problems with the wording might be a possible explanation, or more likely the composition of the respective language sample. The Swedish sample contained the most severely impaired patients (GCS), with the lowest functional level of recovery (GOSE) and the highest injury severity score (AIS). Thus, individuals in the Swedish sample seem to be more severely injured compared to other language samples. Therefore, the low correlation of this PHQ-9 item may be attributable to the particularities of the Swedish sample. Future research could review the wording of this item and examine the Swedish PHQ-9 in a broader spectrum of TBI severities.

Additionally, one item from the PCL-5 (“*Trouble remembering important parts of the stressful experience*”) did not distinguish well between low and high levels of PTSD across all languages and displayed low correlations with the scale score (i.e., DSM-5 cluster) in French, Norwegian, and Swedish translations. The factorial structure of the original PCL-5 has been examined on several occasions [74,75], whereby this item was re-assigned to different dimensions. The results of the present study indicate that PCL-5 translations have adopted the methodological problem of the original questionnaire version. Thus, further investigation of the factorial structure of the PCL-5 could lead to an amelioration of the questionnaire’s psychometric characteristics.

As expected, the validity inspection of the PROMs (newly translated and available prior to the CENTER-TBI study) indicated medium to strong correlations with the SF-36v2, the SF-12v2, and the GOSE in most languages. The PCS and MCS of the SF-36v2 and SF-12v2 generally demonstrated negatively medium to strong negative correlations with the GAD-7, PHQ-9, PCL-5, and RPQ. One exception was the GAD-7, which revealed a low correlation with the PCS of the SF-36v2 in English and Swedish and the PCS of the SF-12v2 in six out of nine languages (Dutch, English, French, Italian, Norwegian, and Swedish). This might be attributable to the items of the SF-12v2 constituting the PCS in the original version. While the items of the SF-36v2 cover a wider range of physical activities and activity-related problems, the items of the SF-12v2 focus on a limited number of physical problems that are most probably associated less with anxiety. Nevertheless, the results are generally in line with previous findings suggesting that negative emotions (i.e., anxiety, depression, or stress) are highly correlated with generic HRQoL, especially with the MCS [76,77]. Moreover, the assumption that the mental and physical components of the SF-36v2 and the SF-12v2 would have strong positive correlations with the QOLIBRI and the QOLIBRI-OS was affirmed across all languages, supporting results from previous studies [34,78].

Generally, the GAD-7 (except for the German language sample), the PHQ-9, the PCL-5 (except for the German language sample), and the RPQ exhibited medium to strong negative correlations with the GOSE. The German individuals after TBI had a relatively high recovery rate with 50% of full recovery after six months (i.e., GOSE = 8); they suffered a less severe TBI (50% had GCS of 15) and were, consequently, less impaired, as reflected by the low correlation. These results are in line with previous research showing that the functional recovery status after TBI is frequently associated with the absence of mental health problems [79] and post-concussion symptoms [80], and vice versa. The GOSE also revealed medium to strong positive correlations with the QOLIBRI and QOLIBRI-OS across all languages, indicating that higher disease-specific HRQoL is associated with better functional outcomes, which is in line with previous research findings [34,38,81].

Further, the TBI severity as assessed by the GCS rating the degree of consciousness displayed a low association with both the psychological and health-related PROMs in almost all languages except for the Swedish translations of the PHQ-9, the RPQ, the QOLIBRI, and the QOLIBRI-OS. Previously published validity results in the field of TBI [59] found no association between GCS and psychological outcomes and post-concussion symptoms. These populations contained a lower number of more severely injured individuals, as measured by the ISS, and therefore, smaller or no correlations were found. In the Swedish translations, the higher association of the GCS and the outcomes in the Swedish sample might be explained by the higher injury severity and stronger polytrauma of the participants.

Overall, the original factorial structures suggested by the instrument developers were replicated for the GAD-7, the PHQ-9, and the QOLIBRI-OS. The translations of the PCL-5 displayed an acceptable model fit, indicating that the initial factorial structure describes the data well. Nevertheless, we recommend a further investigation of the item “*Trouble remembering important parts of the stressful experience”* which displayed irregularities in both reliability analyses across all languages and the factor loadings of the CFA in some translations. The five-factor structure of the original QOLIBRI was replicated in all but two language samples; the English and Finnish models did not converge. This could be due to several reasons: extreme response categories rarely chosen by the participants, relatively large number of parameters that must be estimated in relation to the sample size, or (unconsidered) correlations between latent factors [61].

The original factor solutions could not be replicated for the RPQ translations; this is in line with previous research findings, as several factor solutions have been proposed for the RPQ [30,31,72,73]. Since the RPQ has primarily been developed for TBI populations, further investigation of the factorial structure and thus implementation of an appropriate scoring are strongly recommended.

The SF-36v2 (except for the Dutch version showing an acceptable model fit) and the SF-12v2 presented a poor model fit. Neither of these instruments were specifically developed for populations after TBI, and they use a wide range of different response scales formats, which might be confusing and tiring, especially for respondents with cognitive deficits, and affect their response behavior [82] resulting in less good fit of the estimated models [83,84]. For the assessment of generic HRQoL in TBI populations, further investigation of the factorial structure of both PROMs seems appropriate.

*Objectivity*. The layout and instructions for administering the newly translated PROMs were internationally harmonized and are therefore similar across all language versions. Moreover, instructions for the assessment, scoring, and interpretation were provided (see the SOPs of the CENTER-TBI study). For the interpretation of results, general population-based norms or reference values are helpful. For example, for the QOLIBRI, population-based reference values for the UK and the Netherlands have recently been made available [35].

*Strengths and limitations of the study*. The main strength of the present study is the broad overview of the psychometric properties of the various previous and newly translated and linguistically validated PROMs in the TBI field [20], which had not yet been carried out.

The psychometric results allow researchers and clinicians to rate the quality of the translated questionnaires before selecting them for national and international studies and clinical practice to evaluate outcomes after TBI.

Because of the small sample sizes in some languages, further modern test theoretical analyses cannot be reported here. Additional research concerning the assumption of measurement invariance (MI) across languages could increase the quality of the instruments even further with respect to the international administration and pooling of international data. MI analysis evaluates whether the same construct is understood and measured across different languages. Some of our recent studies have already shown that the PHQ-9 and GAD-7 [85], QOLIBRI [35], and QOLIBRI-OS [39] applied in the field of TBI measure one and the same construct across languages. Furthermore, follow-up studies will focus on assessing measurement invariance comparisons of the different constructs in the individual PROMs in the different languages and the sensitivity and responsiveness of the PROMs for different patient groups and risk factors.

The present study also has some limitations. Despite the large number of participants in the CENTER-TBI core study, the psychometric properties of some translations could not be examined because of the limited number of participants. Consequently, the Danish, Hebrew, Hungarian, Latvian, Lithuanian, Romanian, and Serbian translations of the PROMs need further investigation with a larger number of patients. Furthermore, given the range of TBI severity (mild to severe) covered, we observed that even six months after TBI, participants with higher TBI severity with and without extracranial injuries and polytrauma were not always able to complete the PROMs. To provide robust psychometric analyses in more severe patient groups, future assessments should be also conducted at later time points.

## 5. Conclusions

This study provides psychometric characteristics of the PROMs administered in the CENTER-TBI study for individuals after TBI. The psychometric properties of these PROMs are satisfactory to excellent on the scale level in nine European languages. These results highlight the value of a rigid process of translation and linguistic and cultural adaptation of questionnaires that goes far beyond a literal translation and that ensures the cultural comparability of the translated versions. Therefore, researchers and clinicians can now select reliable and valid instruments for clinical use, data collection, and aggregation, when evaluating outcomes after TBI in international studies, thus improving outcome assessment in national and international healthcare.

## Figures and Tables

**Table 1:** Reliability of the GAD-7: Comparison of the CENTER-TBI results with the values from the original English validation study and the first English validation study in the field of TBI.

GAD-7	CENTER-TBI ^1^									Original English Version ^2^	
	Dutch	English	Finnish	French	German	Italian	Norwegian	Spanish	Swedish	Non-TBI	TBI
*N*	584	214	207	109	78	266	253	253	60	2740	1838
Cronbach’s alpha	**0.92**	**0.90**	**0.86**	**0.94**	**0.84**	**0.92**	**0.90**	**0.89**	**0.86**	**0.92**	**0.88**
*N*	584	214	207	109	78	266	253	253	60	591	-
Split-half or test–retest reliability ^3^	**0.93**	**0.91**	**0.89**	**0.95**	**0.85**	**0.95**	**0.93**	**0.93**	**0.83**	**0.83**	-

Note. ^1^ Reliability coefficients obtained from the CENTER-TBI study sample. ^2^ Reliability coefficients from the original English validation of the GAD-7 in a non-TBI sample [23], and from the first English validation in a TBI sample [68]. ^3^ Split-half reliability (CENTER-TBI data), test–retest reliability provided by original studies; *N* = number of cases; values in **bold** represent at least satisfactory reliability (≥0.70).

**Table 2:** Reliability of the PHQ-9: Comparison of the CENTER-TBI results with the values from the original English validation study and the first English validation study in the field of TBI.

PHQ-9	CENTER-TBI ^1^									Original English Version ^2^	
	Dutch	English	Finnish	French	German	Italian	Norwegian	Spanish	Swedish	Non-TBI	TBI
*N*	587	214	206	107	81	265	254	253	60	3000	168 ^†^
Cronbach’s alpha	**0.88**	**0.88**	**0.86**	**0.87**	**0.85**	**0.89**	**0.89**	**0.88**	**0.78**	**0.89**	**0.91** ^†^
*N*	587	214	206	107	81	265	254	253	60	580	132 ^‡^
Split-half or test–retest reliability ^3^	**0.90**	**0.90**	**0.92**	**0.91**	**0.90**	**0.91**	**0.91**	**0.91**	**0.85**	**0.84**	**0.76** ^‡^

Note. ^1^ Reliability coefficients (CENTER-TBI study sample). ^2^ Reliability coefficients (original English validation of the PHQ-9 in a non-TBI sample [24] and from two English validations in TBI samples) ^†^ Cronbach’s alpha [69] and ^‡^ test–retest reliability [70]. ^3^ Split-half reliability (CENTER-TBI data), test–retest reliability provided by original studies; *N* = number of cases; values in **bold** represent at least satisfactory reliability (≥0.70).

**Table 3:** Reliability of the PCL-5: Comparison of the CENTER-TBI results with the values from the original English validation study.

PCL-5	DSM-5 Cluster	CENTER-TBI ^1^									Original English Version ^2^	
		Dutch *	English	Finnish *	French *	German *	Italian *	Norwegian	Spanish *	Swedish *	Non-TBI	TBI
*N*	-	586	213	212	110	76	261	248	256	57	278 ^†^	-
Cronbach’s alpha	B	**0.90**	**0.89**	**0.81**	**0.89**	**0.88**	**0.90**	**0.90**	**0.88**	**0.88**	**0.80**	**-**
	C	**0.79**	**0.83**	**0.78**	**0.92**	**0.77**	**0.90**	**0.82**	**0.82**	**0.82**	**0.83**	
	D	**0.85**	**0.84**	**0.79**	**0.83**	**0.78**	**0.83**	**0.79**	**0.87**	**0.80**	**0.82**	
	E	**0.79**	**0.81**	**0.83**	**0.83**	**0.74**	**0.83**	**0.82**	**0.84**	**0.76**	**0.75**	
	Total	**0.93**	**0.94**	**0.92**	**0.93**	**0.92**	**0.95**	**0.93**	**0.94**	**0.91**	**0.94** ^†^**/0.92**	
*N*	-	586	213	212	110	76	261	248	256	57	53/912	-
Split-half or test–retest reliability ^3^	B	**0.92**	**0.91**	**0.86**	**0.91**	**0.91**	**0.92**	**0.92**	**0.88**	**0.84**	**0.80**	**-**
	C	**-**	**-**	**-**	**-**	**-**	**-**	**-**	**-**	**-**	**0.83**	
	D	**0.87**	**0.87**	**0.83**	**0.84**	**0.86**	**0.85**	**0.83**	**0.89**	**0.85**	**0.82**	
	E	**0.84**	**0.85**	**0.84**	**0.85**	**0.78**	**0.85**	**0.86**	**0.90**	**0.88**	**0.75**	
	Total	**0.96**	**0.95**	**0.94**	**0.94**	**0.92**	**0.95**	**0.95**	**0.96**	**0.92**	**0.82** ^†^**/0.91**	

Note. * Instruments translated and linguistically validated for the CENTER-TBI study. ^1^ Reliability coefficients (CENTER-TBI study sample). ^2^ Reliability coefficients (original English validation of the PCL-5 in a non-TBI sample on the ^†^ total score level [26]) and on the total score and cluster level [71]. ^3^ Split-half reliability (CENTER-TBI data), test–retest reliability provided by original studies; DSM-5 clusters: B = Intrusion; C = Avoidance; D = Negative alterations in cognition and mood; E = Hyperarousal; Cronbach’s alpha and split-half reliability not reported due to the scale length (two items); *N* = number of cases; values in **bold** represent at least satisfactory reliability (≥0.70).

**Table 4:** Reliability of the RPQ: Comparison of the CENTER-TBI results with the values from the original English validation study in the field of TBI.

RPQ	CENTER-TBI ^1^									Original English Version ^2^
	Dutch *	English	Finnish *	French *	German	Italian *	Norwegian	Spanish *	Swedish *	TBI
*N*	597	223	213	115	80	268	263	254	59	41
Cronbach’s alpha	**0.93**	**0.92**	**0.92**	**0.92**	**0.89**	**0.91**	**0.92**	**0.92**	**0.89**	**-**
*N*	597	223	213	115	80	268	263	254	59	-
Split-half or test–retest reliability ^3^	**0.94**	**0.95**	**0.95**	**0.94**	**0.93**	**0.94**	**0.93**	**0.92**	**0.82**	**0.90**

Note. * Instruments translated and linguistically validated for the CENTER-TBI study. ^1^ Reliability coefficients (CENTER-TBI study sample). ^2^ Reliability coefficients from the original English validation of the RPQ in a TBI sample [30]. ^3^ Split-half reliability (CENTER-TBI data), test–retest reliability (original validation study); *N* = number of cases; values in **bold** represent at least satisfactory reliability (≥0.70).

**Table 5:** Reliability of the QOLIBRI: Comparison of the CENTER-TBI results with the values from the original English validation study in the field of TBI.

QOLIBRI	Scale	CENTER-TBI ^1^									Original English Version ^2^
		Dutch	English	Finnish	French	German	Italian	Norwegian	Spanish	Swedish	TBI
*N*	-	583	224	207	104	77	271	247	255	57	97
Cronbach’s alpha	Cognition	**0.92**	**0.92**	**0.92**	**0.93**	**0.91**	**0.93**	**0.93**	**0.92**	**0.92**	**0.92**
	Self	**0.92**	**0.92**	**0.94**	**0.94**	**0.92**	**0.94**	**0.93**	**0.93**	**0.92**	**0.90**
	Daily life	**0.93**	**0.93**	**0.92**	**0.93**	**0.93**	**0.94**	**0.90**	**0.94**	**0.92**	**0.93**
	Social	**0.86**	**0.88**	**0.85**	**0.89**	**0.87**	**0.88**	**0.84**	**0.84**	**0.76**	**0.88**
	Emotions	**0.87**	**0.85**	**0.82**	**0.89**	**0.86**	**0.86**	**0.85**	**0.86**	**0.86**	**0.88**
	Physical	**0.79**	**0.79**	**0.79**	**0.81**	**0.76**	**0.85**	**0.82**	**0.84**	**0.75**	**0.80**
	Total	**0.96**	**0.96**	**0.96**	**0.97**	**0.96**	**0.97**	**0.96**	**0.96**	**0.95**	**0.97**
*N*	-	583	224	207	104	77	271	247	255	57	56
Split-half or test–retest reliability ^3^	Cognition	**0.90**	**0.91**	**0.93**	**0.94**	**0.92**	**0.94**	**0.92**	**0.91**	**0.92**	**0.80**
	Self	**0.93**	**0.94**	**0.96**	**0.95**	**0.92**	**0.95**	**0.94**	**0.94**	**0.93**	**0.83**
	Daily life	**0.95**	**0.95**	**0.94**	**0.94**	**0.95**	**0.94**	**0.92**	**0.95**	**0.96**	**0.77**
	Social	**0.88**	**0.91**	**0.86**	**0.95**	**0.90**	**0.89**	**0.86**	**0.89**	**0.86**	**0.79**
	Emotions	**0.89**	**0.86**	**0.87**	**0.90**	**0.89**	**0.88**	**0.88**	**0.89**	**0.88**	**0.76**
	Physical	**0.78**	**0.76**	**0.78**	**0.80**	**0.83**	**0.88**	**0.83**	**0.86**	**0.77**	**0.83**
	Total	**0.97**	**0.98**	**0.98**	**0.98**	**0.98**	**0.98**	**0.98**	**0.97**	**0.98**	**0.88**

Note. ^1^ Reliability coefficients (CENTER-TBI study sample). ^2^ Reliability coefficients (original English validation of the QOLIBRI in a TBI sample [32]). ^3^ Split-half reliability (CENTER-TBI data), test–retest reliability (original study); *N* = number of cases; values in **bold** represent at least satisfactory reliability (≥0.70).

**Table 6:** Comparison of the CENTER-TBI results with the values from the original English validation study in the field of TBI.

QOLIBRI-OS	CENTER-TBI ^1^									Original English Version ^2^
	Dutch	English	Finnish	French	German	Italian	Norwegian	Spanish	Swedish	TBI
*N*	602	239	227	109	84	280	261	265	63	97
Cronbach’s alpha	**0.91**	**0.90**	**0.92**	**0.90**	**0.88**	**0.91**	**0.92**	**0.90**	**0.91**	**0.91**
*N*	602	239	227	109	84	280	261	265	63	54
Split-half or test–retest reliability ^3^	**0.92**	**0.92**	**0.94**	**0.94**	**0.90**	**0.92**	**0.92**	**0.93**	**0.93**	0.69

Note. ^1^ Reliability coefficients (CENTER-TBI study sample). ^2^ Reliability coefficients (original English validation of the QOLIBRI-OS in a TBI sample [38]). ^3^ Split-half reliability (CENTER-TBI data), test–retest reliability (original study); *N* = number of cases; values in **bold** represent at least satisfactory reliability (≥0.70).

**Table 7:** Reliability of the SF-36v2: Comparison of the CENTER-TBI results with the values from the original English validation study.

SF-36v2	Scale	CENTER-TBI ^1^									Original English Version ^2^	
		Dutch	English	Finnish	French	German	Italian	Norwegian	Spanish	Swedish	Non-TBI	TBI
*N*	-	579	220	214	110	78	270	254	255	57	4024–4036	-
Cronbach’s alpha	PF	**0.94**	**0.94**	**0.93**	**0.94**	**0.92**	**0.95**	**0.93**	**0.94**	**0.95**	**0.94**	**-**
	RP	**0.95**	**0.95**	**0.94**	**0.95**	**0.95**	**0.96**	**0.96**	**0.96**	**0.95**	**0.96**	
	BP	**0.89**	**0.87**	**0.88**	**0.88**	**0.89**	**0.88**	**0.87**	**0.87**	**0.86**	**0.87**	
	GH	**0.81**	**0.81**	**0.84**	**0.84**	**0.77**	**0.81**	**0.79**	**0.79**	**0.73**	**0.82**	
	VT	**0.83**	**0.87**	**0.88**	**0.85**	**0.85**	**0.86**	**0.86**	**0.83**	**0.88**	**0.87**	
	SF	**0.82**	**0.87**	**0.87**	**0.90**	**0.81**	**0.82**	**0.87**	**0.87**	**0.88**	**0.84**	
	RE	**0.94**	**0.90**	**0.91**	**0.94**	**0.92**	**0.94**	**0.95**	**0.94**	**0.93**	**0.93**	
	MH	**0.87**	**0.83**	**0.87**	**0.89**	**0.85**	**0.87**	**0.85**	**0.86**	**0.89**	**0.87**	
	HT	**-**	**-**	**-**	**-**	**-**	**-**	**-**	**-**	**-**	**-**	
	PCS	**0.94**	**0.94**	**0.94**	**0.94**	**0.93**	**0.95**	**0.93**	**0.95**	**0.94**	**0.96**	
	MCS	**0.94**	**0.93**	**0.94**	**0.95**	**0.94**	**0.94**	**0.94**	**0.94**	**0.94**	**0.93**	
*N*	-	579	220	214	110	78	270	254	255	57	147	-
Split-half or test–retest reliability ^3^	PF	**0.97**	**0.97**	**0.97**	**0.97**	**0.95**	**0.98**	**0.96**	**0.97**	**0.97**	**0.85**	**-**
	RP	**0.96**	**0.94**	**0.95**	**0.95**	**0.97**	**0.97**	**0.97**	**0.97**	**0.97**	**0.78**	
	BP	**-**	**-**	**-**	**-**	**-**	**-**	**-**	**-**	**-**	**0.71**	
	GH	**0.72**	0.69	**0.78**	**0.75**	0.65	**0.74**	0.68	0.67	0.59	**0.87**	
	VT	**0.85**	**0.90**	**0.94**	**0.95**	**0.89**	**0.89**	**0.89**	**0.91**	**0.92**	**0.75**	
	SF	**-**	**-**	**-**	**-**	**-**	**-**	**-**	**-**	**-**	**0.70**	
	RE	**0.95**	**0.93**	**0.91**	**0.96**	**0.95**	**0.95**	**0.95**	**0.93**	**0.93**	0.61	
	MH	**0.86**	**0.82**	**0.86**	**0.84**	**0.86**	**0.85**	**0.81**	**0.83**	**0.92**	**0.76**	
	HT	**-**	**-**	**-**	**-**	**-**	**-**	**-**	**-**	**-**	**-**	
	PCS	**0.96**	**0.96**	**0.97**	**0.97**	**0.94**	**0.97**	**0.95**	**0.97**	**0.96**	**0.88**	
	MCS	**0.96**	**0.95**	**0.96**	**0.97**	**0.97**	**0.97**	**0.96**	**0.95**	**0.98**	**0.79**	

Note. ^1^ Reliability coefficients (CENTER-TBI study sample). ^2^ Reliability coefficients (original English validation of the QOLIBRI in a non-TBI sample [41]). ^3^ Split-half reliability (CENTER-TBI data), test–retest reliability (original validation study); Cronbach’s alpha and split-half reliability are not reported due to the scale length (two items); PF = Physical functioning; BP = Bodily Pain; GH = General Health; VT = Vitality; SF = Social Functioning; RE = Role-Emotional; MH = Mental Health; HT = Reported Health Transition; PCS = Physical Component Score; MCS = Mental Component Score; split-half reliability not reported for the BP and SF scales due to the scale length (two items); no psychometric properties reported for the HT scale due to the scale length (one item); *N* = number of cases; values in **bold** represent at least satisfactory reliability (≥0.70).

**Table 8:** Reliability of the SF-12v2: Comparison of the CENTER-TBI results with the values from the original English validation study.

SF-12v2	Component Score	CENTER-TBI ^1^						Original English Version ^2^	
		Dutch	English	Finnish	German	Italian	Spanish	Non-TBI	TBI
*N*	-	241	54	172	68	138	210	4002	-
Cronbach’s alpha	PCS	**0.86**	**0.91**	**0.90**	**0.89**	**0.89**	**0.91**	**0.92**	**-**
	MCS	**0.89**	**0.90**	**0.86**	**0.94**	**0.89**	**0.89**	**0.88**	
*N*	-	241	54	172	68	138	210	215	-
Split-half or test–retest reliability ^3^	PCS	**0.91**	**0.95**	**0.92**	**0.92**	**0.94**	**0.95**	**0.85**	**-**
	MCS	**0.89**	**0.90**	**0.88**	**0.92**	**0.89**	**0.92**	0.67	

Note. French, Norwegian, and Swedish language samples were excluded from the reliability analyses due to the low number of participants (*N* < 50). ^1^ Reliability coefficients (CENTER-TBI study sample). ^2^ Reliability coefficients (original English validation of the SF-12v2 in a non-TBI sample [42]). ^3^ Split-half reliability (CENTER-TBI study), test–retest reliability (original validation study); *N* = number of cases; PCS = Physical Component Score; MCS = Mental Component Score; values in **bold** represent at least satisfactory reliability (≥0.70).

**Table 9:** Convergent and discriminant validity of the GAD-7, PHQ-9, PCL-5, RPQ, QOLIBRI, and QOLIBRI-OS with the SF-36v2, the SF-12v2, the GOSE, and the GCS.

		Convergent Validity					Discriminant Validity
Instrument	Language/Value	SF-36v2 PCS	SF-36v2 MCS	SF-12v2 PCS	SF-12v2 MCS	GOSE	GCS
GAD-7	Dutch	**−0.31**	**−0.71**	−0.27	**−0.70**	**−0.41**	−0.11
	English	−0.15	**−0.76**	−0.15	**−0.71**	**−0.36**	−0.04
	Finnish	**−0.35**	**−0.73**	**−0.34**	**−0.69**	**−0.52**	−0.20
	French	**−0.33**	**−0.78**	−0.26	**−0.74**	**−0.35**	−0.09
	German	**−0.45**	**−0.74**	**−0.31**	**−0.72**	−0.24	0.01
	Italian	**−0.31**	**−0.77**	−0.27	**−0.74**	**−0.30**	0.06
	Norwegian	**−0.33**	**−0.74**	−0.29	**−0.72**	**−0.32**	0.07
	Spanish	**−0.38**	**−0.72**	**−0.40**	**−0.68**	**−0.39**	−0.04
	Swedish	−0.12	**−0.65**	−0.22	**−0.63**	**−0.54**	**−0.30**
	*M*	−0.30	−0.73	−0.28	−0.70	−0.38	−0.07
	*Max*	−0.12	−0.65	−0.15	−0.63	−0.24	0.07
	*Min*	−0.45	−0.78	−0.40	−0.74	−0.54	−0.30
	*SD*	0.10	0.04	0.07	0.04	0.10	0.12
PHQ-9	Dutch	**−0.46**	**−0.74**	**−0.43**	**−0.71**	**−0.49**	−0.14
	English	**−0.33**	**−0.77**	**−0.32**	**−0.74**	**−0.47**	−0.13
	Finnish	**−0.47**	**−0.77**	**−0.50**	**−0.71**	**−0.56**	−0.07
	French	**−0.39**	**−0.83**	**−0.36**	**−0.79**	**−0.41**	−0.19
	German	**−0.60**	**−0.61**	**−0.56**	**−0.68**	**−0.45**	0.06
	Italian	**−0.45**	**−0.73**	**−0.43**	**−0.70**	**−0.41**	−0.01
	Norwegian	**−0.44**	**−0.76**	**−0.38**	**−0.76**	**−0.37**	0.02
	Spanish	**−0.49**	**−0.76**	**−0.49**	**−0.74**	**−0.44**	−0.04
	Swedish	**−0.43**	**−0.68**	**−0.52**	**−0.67**	**−0.63**	**−0.33**
	*M*	−0.45	−0.74	−0.44	−0.72	−0.47	−0.09
	*Max*	−0.33	−0.61	−0.32	−0.67	−0.37	0.06
	*Min*	−0.60	−0.83	−0.56	−0.79	−0.63	−0.33
	*SD*	0.07	0.06	0.08	0.04	0.08	0.12
PCL-5	Dutch *	**−0.39**	**−0.63**	**−0.36**	**−0.62**	**−0.45**	−0.20
	English	**−0.29**	**−0.71**	−0.28	**−0.66**	**−0.44**	−0.14
	Finnish *	**−0.38**	**−0.66**	**−0.40**	**−0.61**	**−0.49**	−0.20
	French *	−0.25	**−0.69**	−0.20	**−0.65**	**−0.31**	−0.07
	German *	**−0.44**	**−0.68**	**−0.37**	**−0.62**	−0.16	0.17
	Italian *	**−0.35**	**−0.71**	**−0.32**	**−0.67**	**−0.33**	0.07
	Norwegian	**−0.42**	**−0.66**	**−0.37**	**−0.65**	**−0.42**	−0.06
	Spanish *	**−0.32**	**−0.61**	**−0.33**	**−0.55**	**−0.44**	−0.10
	Swedish *	−0.30	**−0.54**	**−0.37**	**−0.48**	**−0.52**	−0.24
	*M*	−0.35	−0.65	−0.33	−0.61	−0.40	−0.09
	*Max*	−0.25	−0.54	−0.20	−0.48	−0.16	0.17
	*Min*	−0.44	−0.71	−0.40	−0.67	−0.52	−0.24
	*SD*	0.06	0.05	0.06	0.06	0.11	0.13
RPQ	Dutch *	**−0.48**	**−0.64**	**−0.45**	**−0.62**	**−0.54**	−0.19
	English	**−0.43**	**−0.63**	**−0.47**	**−0.62**	**−0.60**	−0.26
	Finnish *	**−0.54**	**−0.60**	**−0.55**	**−0.54**	**−0.63**	−0.19
	French *	**−0.44**	**−0.71**	**−0.40**	**−0.67**	**−0.39**	−0.07
	German	**−0.50**	**−0.44**	**−0.47**	**−0.46**	**−0.52**	−0.07
	Italian *	**−0.43**	**−0.62**	**−0.44**	**−0.56**	**−0.47**	−0.07
	Norwegian	**−0.51**	**−0.59**	**−0.47**	**−0.57**	**−0.58**	−0.11
	Spanish *	**−0.52**	**−0.61**	**−0.52**	**−0.56**	**−0.63**	−0.21
	Swedish *	**−0.38**	**−0.45**	**−0.44**	**−0.42**	**−0.59**	**−0.34**
	*M*	−0.47	−0.59	−0.47	−0.56	−0.55	−0.17
	*Max*	−0.38	−0.44	−0.40	−0.42	−0.39	−0.07
	*Min*	−0.54	−0.71	−0.55	−0.67	−0.63	−0.34
	*SD*	0.05	0.09	0.04	0.08	0.08	0.09
QOLIBRI	Dutch	**0.58**	**0.71**	**0.56**	**0.69**	**0.54**	0.23
	English	**0.51**	**0.74**	**0.53**	**0.71**	**0.60**	0.21
	Finnish	**0.59**	**0.80**	**0.59**	**0.72**	**0.59**	0.12
	French	**0.53**	**0.76**	**0.49**	**0.74**	**0.53**	0.11
	German	**0.62**	**0.68**	**0.56**	**0.68**	**0.37**	−0.09
	Italian	**0.55**	**0.74**	**0.56**	**0.71**	**0.52**	0.00
	Norwegian	**0.53**	**0.73**	**0.51**	**0.71**	**0.44**	−0.01
	Spanish	**0.65**	**0.63**	**0.65**	**0.61**	**0.56**	0.16
	Swedish	**0.59**	**0.66**	**0.60**	**0.61**	**0.64**	**0.34**
	*M*	0.57	0.72	0.56	0.69	0.53	0.12
	*Max*	0.65	0.80	0.65	0.74	0.64	0.34
	*Min*	0.51	0.63	0.49	0.61	0.37	−0.09
	*SD*	0.05	0.05	0.05	0.05	0.08	0.14
QOLIBRI-OS	Dutch	**0.58**	**0.65**	**0.57**	**0.66**	**0.50**	0.19
	English	**0.49**	**0.72**	**0.52**	**0.72**	**0.53**	0.17
	Finnish	**0.51**	**0.74**	**0.55**	**0.66**	**0.48**	−0.01
	French	**0.63**	**0.66**	**0.60**	**0.62**	**0.56**	0.25
	German	**0.63**	**0.57**	**0.55**	**0.67**	**0.40**	−0.08
	Italian	**0.57**	**0.65**	**0.59**	**0.61**	**0.44**	−0.04
	Norwegian	**0.49**	**0.69**	**0.48**	**0.66**	**0.47**	0.01
	Spanish	**0.59**	**0.58**	**0.61**	**0.58**	**0.55**	0.19
	Swedish	**0.57**	**0.61**	**0.59**	**0.61**	**0.62**	**0.40**
	*M*	0.56	0.65	0.56	0.64	0.51	0.12
	*Max*	0.63	0.74	0.61	0.72	0.62	0.40
	*Min*	0.49	0.57	0.48	0.58	0.40	−0.08
	*SD*	0.05	0.06	0.04	0.04	0.07	0.16

Note. * Instrument translated and linguistically validated for the CENTER-TBI study; *M* = mean, *Max* = maximum, *Min* = minimum; *SD* = standard deviation; SF-36v2-PCS = physical component score; SF-36v2—MCS = mental component score; SF-12v2—PCS = physical component score SF-12v2—MCS = mental component score.; GCS = Glasgow Coma Scale; GOSE = Glasgow Outcome Scale—Extended. Values in **bold** represent an at least medium effect size (≥|0.30|), significant at α = 0.05.

**Table 10:** Factorial validity: results of the CFA.

Instrument	Language	χ^2^	*df*	*p*	CFI	TLI	RMSEA	90% CI	SRMR
GAD-7	Dutch	27.90	14	0.015	**1.00**	**1.00**	**0.04**	**[0.02,0.06]**	**0.03**
	English	38.69	14	<0.001	**1.00**	**0.99**	0.09	[0.06,0.13]	**0.06**
	Finnish	15.82	14	**0.325**	**1.00**	**1.00**	**0.03**	[0.00,0.07]	**0.05**
	Italian	49.29	14	<0.001	**1.00**	**1.00**	0.10	[0.07,0.13]	**0.05**
	Norwegian	9.28	14	**0.813**	**1.00**	**1.00**	**0.00**	**[0.00,0.04]**	**0.03**
	Spanish	57.87	14	<0.001	**0.99**	**0.99**	0.11	[0.08,0.14]	**0.06**
PHQ-9	Dutch	54.56	27	0.001	**1.00**	**1.00**	**0.04**	[0.03,0.06]	**0.05**
	English	46.62	27	**0.011**	**1.00**	**0.99**	**0.06**	[0.03,0.09]	**0.07**
	Finnish	54.38	27	0.001	**0.99**	**0.99**	0.07	[0.04,0.10]	0.08
	Italian	17.65	27	**0.914**	**1.00**	**1.00**	**0.00**	**[0.00,0.02]**	**0.04**
	Norwegian	18.63	27	**0.883**	**1.00**	**1.00**	**0.00**	**[0.00,0.02]**	**0.04**
	Spanish	42.95	27	**0.026**	**1.00**	**1.00**	**0.05**	[0.02,0.08]	**0.06**
PCL-5	Dutch *	264.37	164	<0.001	**1.00**	**1.00**	**0.03**	**[0.03,0.04]**	**0.05**
	English	241.20	164	<0.001	**1.00**	**1.00**	**0.05**	[0.03,0.06]	**0.07**
	Finnish *	NA	NA	NA	NA	NA	NA	NA	NA
	Italian *	305.80	164	<0.001	**1.00**	**1.00**	**0.06**	[0.05,0.07]	**0.07**
	Norwegian	201.45	164	0.025	**1.00**	**1.00**	**0.03**	**[0.01,0.04]**	**0.06**
	Spanish *	373.92	164	<0.001	**0.99**	**0.99**	0.07	[0.06,0.08]	**0.07**
RPQ	Dutch *	786.30	104	<0.001	**0.99**	**0.99**	0.11	[0.10,0.11]	**0.08**
	English	345.83	104	<0.001	**0.99**	**0.98**	0.10	[0.09,0.12]	0.09
	Finnish *	280.16	104	<0.001	**0.99**	**0.99**	0.09	[0.08,0.10]	0.09
	Italian *	429.59	104	<0.001	**0.98**	**0.98**	0.11	[0.10,0.12]	0.09
	Norwegian	230.42	104	<0.001	**0.99**	**0.99**	0.07	[0.06,0.08]	**0.08**
	Spanish *	285.93	104	<0.001	**0.98**	**0.98**	0.08	[0.07,0.10]	0.09
QOLIBRI	Dutch	1299.37	614	<0.001	**1.00**	**1.00**	**0.05**	**[0.04,0.05]**	**0.05**
	English	NA	NA	NA	NA	NA	NA	NA	NA
	Finnish	NA	NA	NA	NA	NA	NA	NA	NA
	Italian	932.84	614	<0.001	**1.00**	**1.00**	**0.05**	**[0.04,0.05]**	**0.05**
	Norwegian	793.74	614	<0.001	**1.00**	**1.00**	**0.04**	**[0.03,0.04]**	**0.06**
	Spanish	889.15	614	<0.001	**1.00**	**1.00**	**0.04**	**[0.04,0.05]**	**0.06**
QOLIBRI-OS	Dutch	37.15	9	<0.001	**1.00**	**1.00**	0.07	[0.05,0.10]	**0.03**
	English	15.22	9	**0.085**	**1.00**	**1.00**	**0.05**	[0.00,0.10]	**0.04**
	Finnish	11.17	9	**0.264**	**1.00**	**1.00**	**0.03**	[0.00,0.09]	**0.03**
	Italian	32.07	9	<0.001	**1.00**	**1.00**	0.10	[0.06,0.13]	**0.04**
	Norwegian	10.39	9	**0.320**	**1.00**	**1.00**	**0.03**	[0.00,0.08]	**0.02**
	Spanish	32.14	9	<0.001	**1.00**	**0.99**	0.10	[0.06,0.14]	**0.04**
SF-36v2	Dutch	2552.88	551	<0.001	**0.99**	**0.99**	0.08	[0.08,0.08]	**0.07**
	English	1239.59	551	<0.001	**0.99**	**0.99**	0.08	[0.07,0.08]	0.09
	Finnish	NA	NA	NA	NA	NA	NA	NA	NA
	Italian	NA	NA	NA	NA	NA	NA	NA	NA
	Norwegian	1300.94	551	<0.001	**1.00**	**0.99**	0.07	[0.07,0.08]	0.09
	Spanish	NA	NA	NA	NA	NA	NA	NA	NA
SF-12v2	Dutch	295.82	53	<0.001	**0.99**	**0.98**	0.14	[0.13,0.16]	0.09
	Finnish	162.88	53	<0.001	**0.99**	**0.99**	0.11	[0.09,0.13]	0.08
	Spanish	113.38	53	<0.001	**1.00**	**1.00**	0.07	[0.06,0.09]	**0.06**

Note. * Instrument translated and linguistically validated for the CENTER-TBI study; χ^2^ = Chi-square statistic, df = degrees of freedom, *p* = *p*-value, CFI = comparative fit index, TLI = Tucker–Lewis index, RMSEA = root mean square error of approximation; 95% CI = 95% confidence interval (lower and upper bound); SRMR = standardized root mean square residual; values in bold indicate satisfactory results according to the respective cut-off values. NA means that the respective model did not converge; no models were estimated for the English, French, German, Italian, Norwegian, and the Swedish SF12-v2 due to the sample size being too small (*N* < 150).

## Data Availability

All relevant data are available upon request from CENTER-TBI, and the authors are not legally allowed to share it publicly. The authors confirm that they received no special access privileges to the data. CENTER-TBI is committed to data sharing and in particular to responsible further use of the data. Hereto, we have a data sharing statement in place: https://www.center-tbi.eu/data/sharing. The CENTER-TBI Management Committee, in collaboration with the General Assembly, established the Data Sharing policy, and Publication and Authorship Guidelines to assure correct and appropriate use of the data as the dataset is hugely complex and requires help of experts from the Data Curation Team or Bio- Statistical Team for correct use. This means that we encourage researchers to contact the CENTER-TBI team for any research plans and the Data Curation Team for any help in appropriate use of the data, including sharing of scripts. Requests for data access can be submitted online: https://www.center-tbi.eu/data. The complete manual for data access is also available online: https://www.center-tbi.eu/files/SOP-Manual-DAPR-20181101.pdf.

## Supplementary Materials

The following materials are available online at https://www.mdpi.com/article/10.3390/jcm10112396/s1. List of the CENTER-TBI participants and investigators; Online Supplement 1 (OS-1: Sociodemographic characteristics); Online Supplement 2 (OS-2: Reliability); Online Supplement 3 (OS-3: Factorial validity), Online Supplement 4 (OS-4: Participants and Investigators).

## Abbreviations

GAD-7	Generalized Anxiety Disorder 7 Item Scale
HRQoL	Health-related quality of life
PHQ-9	Patient Health Questionnaire
PCL-5	Posttraumatic Stress Disorder Checklist-5
RPQ	Rivermead Post-Concussion Symptoms Questionnaire
QOLIBRI	Quality of Life after Traumatic Brain Injury
QOLIBRI-OS	Quality of Life after Traumatic Brain Injury—Overall Scale
SF-36v2	36-item Short Form Health Survey—Version 2
SF-12v2	12-Item Short Form Survey—Version 2
PCS	Physical component score
MCS	Mental component score
MI	Measurement invariance
PF	Physical functioning
RP	Role—Physical
GH	General Health
VT	Vitality
SF	Social functioning
RE	Role—Emotional
MH	Mental health
GOSE	Glasgow Outcome Scale—Extended
GCS	Glasgow Coma Scale

## Appendix A. Number of Participants (GOSE and GCS)

**Table A1:** Number of participants for convergent (assessed with the GOSE) and discriminant (assessed with the GCS) validity per language and outcome instrument.

Instrument	Language	GOSE	GCS
GAD-7	Dutch	**584**	**569**
	English	**213**	**213**
	Finnish	**207**	**199**
	French	**109**	**100**
	German	**78**	**77**
	Italian	**266**	**266**
	Norwegian	**253**	**251**
	Spanish	**253**	**251**
	Swedish	**60**	**57**
PHQ-9	Dutch	**587**	**572**
	English	**213**	**213**
	Finnish	**206**	**198**
	French	**107**	**99**
	German	**81**	**80**
	Italian	**265**	**265**
	Norwegian	**254**	**252**
	Spanish	**253**	**251**
	Swedish	**60**	**57**
PCL-5	Dutch	**586**	**570**
	English	**212**	**212**
	Finnish	**212**	**204**
	French	**110**	**103**
	German	**76**	**75**
	Italian	**261**	**261**
	Norwegian	**248**	**246**
	Spanish	**256**	**254**
	Swedish	**57**	**54**
RPQ	Dutch	**597**	**582**
	English	**222**	**222**
	Finnish	**213**	**205**
	French	**115**	**107**
	German	**80**	**79**
	Italian	**268**	**268**
	Norwegian	**263**	**261**
	Spanish	**254**	**252**
	Swedish	**59**	**56**
QOLIBRI	Dutch	**583**	**568**
	English	**223**	**222**
	Finnish	**207**	**199**
	French	**104**	**96**
	German	**77**	**76**
	Italian	**270**	**271**
	Norwegian	**247**	**245**
	Spanish	**255**	**253**
	Swedish	**57**	**54**
QOLIBRI-OS	Dutch	**602**	**585**
	English	**238**	**238**
	Finnish	**227**	**219**
	French	**109**	**99**
	German	**84**	**83**
	Italian	**279**	**280**
	Norwegian	**261**	**259**
	Spanish	**265**	**263**
	Swedish	**63**	**60**
SF-36v2	Dutch	**579**	**564**
	English	**219**	**218**
	Finnish	**214**	**206**
	French	**110**	**100**
	German	**78**	**77**
	Italian	**269**	**270**
	Norwegian	**254**	**252**
	Spanish	**255**	**253**
	Swedish	**57**	**54**
SF-12v2 *	Dutch	**241**	**229**
	English	**54**	**54**
	Finnish	**172**	**168**
	French	30	27
	German	**68**	**67**
	Italian	**138**	**138**
	Norwegian	32	32
	Spanish	**210**	**209**
	Swedish	15	12
SF-12v2 combined **	Dutch	**605**	**588**
	English	**242**	**241**
	Finnish	**231**	**223**
	French	**110**	**100**
	German	**82**	**81**
	Italian	**275**	**276**
	Norwegian	**259**	**257**
	Spanish	**257**	**255**
	Swedish	**58**	**55**

Note. * Reported SF-12v2 values used for the reliability analyses. ** Combined SF-12v2 values (i.e., reported values and derived from the respective items of the SF-36v2) were used for the convergent and divergent validity analyses; **bold** values represent samples with N ≥ 50.

## Appendix B. Validity on Scale Level

**Table A2:** Convergent and discriminant validity of the PCL-5 scales.

		Convergent Validity					Discriminant Validity
Cluster	Language/Value	SF-36v2 PCS	SF-36v2 MCS	SF-12v2 PCS	SF-12v2 MCS	GOSE	GCS
B	Dutch	**−0.33**	**−0.46**	**−0.32**	**−0.45**	−0.29	−0.15
	English	−0.24	**−0.47**	−0.24	**−0.43**	−0.18	−0.03
	Finnish	**−0.38**	**−0.43**	**−0.33**	**−0.39**	−0.27	−0.09
	French	−0.24	**−0.43**	−0.18	**−0.44**	−0.21	0.02
	German	−0.28	**−0.61**	−0.20	**−0.47**	0.02	0.26
	Italian	−0.30	**−0.57**	−0.27	**−0.53**	−0.24	0.13
	Norwegian	**−0.33**	**−0.49**	−0.29	**−0.49**	−0.27	−0.04
	Spanish	−0.25	**−0.40**	−0.24	**−0.33**	−0.28	0.03
	Swedish	−0.20	−0.27	−0.26	−0.19	**−0.35**	−0.13
Total	*M*	−0.28	−0.46	−0.26	−0.41	−0.23	0.00
	*Max*	−0.20	−0.27	−0.18	−0.19	0.02	0.26
	*Min*	−0.38	−0.61	−0.33	−0.53	−0.35	−0.15
	*SD*	0.06	0.10	0.05	0.10	0.11	0.13
C	Dutch	−0.26	**−0.36**	−0.23	**−0.37**	−0.23	−0.09
	English	−0.18	**−0.38**	−0.19	**−0.34**	−0.12	0.00
	Finnish	−0.27	**−0.42**	−0.26	**−0.39**	−0.28	−0.14
	French	−0.13	**−0.45**	−0.11	**−0.39**	−0.10	0.10
	German	−0.27	**−0.48**	−0.21	**−0.42**	−0.03	0.26
	Italian	−0.19	**−0.48**	−0.17	**−0.46**	−0.15	0.09
	Norwegian	−0.23	**−0.38**	−0.20	**−0.38**	−0.21	−0.03
	Spanish	−0.21	−0.29	−0.17	−0.28	−0.20	0.01
	Swedish	−0.26	−0.24	−0.26	−0.15	−0.26	−0.03
Total	*M*	−0.22	−0.39	−0.20	−0.35	−0.18	0.02
	*Max*	−0.13	−0.24	−0.11	−0.15	−0.03	0.26
	*Min*	−0.27	−0.48	−0.26	−0.46	−0.28	−0.14
	*SD*	0.05	0.08	0.05	0.09	0.08	0.12
D	Dutch	**−0.33**	**−0.57**	**−0.31**	**−0.57**	**−0.41**	−0.22
	English	−0.22	**−0.67**	−0.21	**−0.62**	**−0.45**	−0.19
	Finnish	−0.24	**−0.59**	−0.26	**−0.57**	**−0.46**	−0.18
	French	−0.20	**−0.61**	−0.15	**−0.57**	−0.29	−0.12
	German	**−0.39**	**−0.60**	**−0.33**	**−0.56**	−0.17	0.14
	Italian	**−0.32**	**−0.68**	−0.28	**−0.65**	**−0.34**	−0.01
	Norwegian	**−0.39**	**−0.57**	**−0.34**	**−0.56**	**−0.41**	−0.06
	Spanish	−0.29	**−0.58**	**−0.30**	**−0.54**	**−0.49**	−0.20
	Swedish	−0.25	**−0.51**	−0.29	**−0.44**	**−0.45**	−0.28
Total	*M*	−0.29	−0.60	−0.27	−0.56	−0.39	−0.12
	*Max*	−0.20	−0.51	−0.15	−0.44	−0.17	0.14
	*Min*	−0.39	−0.68	−0.34	−0.65	−0.49	−0.28
	*SD*	0.07	0.05	0.06	0.06	0.10	0.13
E	Dutch	**−0.37**	**−0.61**	**−0.34**	**−0.60**	**−0.43**	−0.17
	English	−0.28	**−0.67**	−0.29	**−0.64**	**−0.36**	−0.09
	Finnish	**−0.42**	**−0.61**	**−0.43**	**−0.55**	**−0.44**	−0.20
	French	−0.25	**−0.70**	−0.21	**−0.66**	**−0.35**	−0.07
	German	**−0.47**	**−0.60**	**−0.42**	**−0.60**	−0.22	0.11
	Italian	**−0.33**	**−0.69**	−0.29	**−0.65**	−0.29	0.12
	Norwegian	**−0.36**	**−0.64**	**−0.31**	**−0.63**	**−0.37**	0.00
	Spanish	**−0.32**	**−0.63**	**−0.33**	**−0.59**	**−0.36**	−0.03
	Swedish	−0.20	**−0.51**	**−0.30**	**−0.48**	**−0.42**	−0.20
Total	*M*	−0.33	−0.63	−0.32	−0.60	−0.36	−0.06
	*Max*	−0.20	−0.51	−0.21	−0.48	−0.22	0.12
	*Min*	−0.47	−0.70	−0.43	−0.66	−0.44	−0.20
	*SD*	0.08	0.06	0.07	0.06	0.07	0.12

Note. Cluster B = Intrusion; Cluster C = Avoidance; Cluster D = Negative alterations in cognition and mood; Cluster E = Hyperarousal; *M* = mean, *Max* = maximum, *Min* = minimum; *SD* = standard deviation; SF-36v2/SF-12v2—PCS = Physical Component Score; SF-36v2/SF-12v2—MCS = Mental Component Score; GCS = Glasgow Coma Scale; GOSE = Glasgow Outcome Scale—Extended. Values in **bold** represent an at least medium effect size (≥|0.30|), significant at *α* = 0.05.

**Table A3:** Convergent and divergent validity of the QOLIBRI scales.

		Convergent Validity					Discriminant Validity
Scale	Language/Values	SF-36v2 PCS	SF-36v2 MCS	SF-12v2 PCS	SF-12v2 MCS	GOSE	GCS
Cognition	Dutch	**0.40**	**0.58**	**0.38**	**0.57**	**0.43**	0.21
	English	**0.34**	**0.61**	**0.37**	**0.58**	**0.43**	0.20
	Finnish	**0.49**	**0.69**	**0.49**	**0.62**	**0.50**	0.07
	French	**0.33**	**0.58**	**0.34**	**0.59**	**0.48**	0.10
	German	**0.46**	**0.53**	**0.39**	**0.52**	0.26	−0.05
	Italian	**0.44**	**0.61**	**0.46**	**0.58**	**0.40**	0.02
	Norwegian	**0.42**	**0.54**	**0.41**	**0.52**	**0.34**	−0.06
	Spanish	**0.41**	**0.52**	**0.43**	**0.49**	**0.33**	0.07
	Swedish	**0.33**	**0.42**	**0.41**	**0.40**	**0.44**	0.21
	*M*	0.40	0.56	0.41	0.54	0.40	0.09
	*Max*	0.49	0.69	0.49	0.62	0.50	0.21
	*Min*	0.33	0.42	0.34	0.40	0.26	−0.06
	*SD*	0.06	0.07	0.05	0.07	0.08	0.11
Self	Dutch	**0.51**	**0.66**	**0.50**	**0.66**	**0.43**	0.19
	English	**0.38**	**0.72**	**0.40**	**0.70**	**0.45**	0.09
	Finnish	**0.50**	**0.78**	**0.52**	**0.69**	**0.50**	0.04
	French	**0.49**	**0.73**	**0.42**	**0.71**	**0.46**	0.07
	German	**0.49**	**0.70**	**0.41**	**0.71**	0.29	−0.15
	Italian	**0.51**	**0.70**	**0.51**	**0.68**	**0.43**	−0.07
	Norwegian	**0.45**	**0.68**	**0.43**	**0.65**	**0.35**	−0.07
	Spanish	**0.57**	**0.61**	**0.61**	**0.59**	**0.46**	0.13
	Swedish	**0.43**	**0.74**	**0.40**	**0.72**	**0.43**	0.06
	*M*	0.48	0.70	0.47	0.68	0.42	0.03
	*Max*	0.57	0.78	0.61	0.72	0.50	0.19
	*Min*	0.38	0.61	0.40	0.59	0.29	−0.15
	*SD*	0.05	0.05	0.07	0.04	0.06	0.11
Daily Life and Autonomy	Dutch	**0.61**	**0.59**	**0.61**	**0.58**	**0.56**	**0.31**
	English	**0.58**	**0.56**	**0.59**	**0.54**	**0.63**	**0.30**
	Finnish	**0.63**	**0.65**	**0.61**	**0.58**	**0.55**	0.14
	French	**0.62**	**0.69**	**0.58**	**0.66**	**0.59**	0.18
	German	**0.63**	**0.58**	**0.55**	**0.58**	**0.41**	0.06
	Italian	**0.64**	**0.65**	**0.64**	**0.61**	**0.62**	0.15
	Norwegian	**0.50**	**0.64**	**0.49**	**0.62**	**0.48**	0.05
	Spanish	**0.70**	**0.48**	**0.69**	**0.46**	**0.54**	0.21
	Swedish	**0.62**	**0.58**	**0.64**	**0.54**	**0.66**	**0.39**
	*M*	0.61	0.60	0.60	0.57	0.56	0.20
	*Max*	0.70	0.69	0.69	0.66	0.66	0.39
	*Min*	0.50	0.48	0.49	0.46	0.41	0.05
	*SD*	0.05	0.06	0.06	0.06	0.08	0.12
Social Relationships	Dutch	0.29	**0.57**	0.30	**0.55**	0.29	0.12
	English	**0.29**	**0.63**	**0.33**	**0.57**	**0.34**	0.06
	Finnish	**0.34**	**0.71**	**0.37**	**0.59**	**0.33**	0.10
	French	**0.35**	**0.61**	**0.32**	**0.58**	**0.33**	0.01
	German	**0.36**	**0.56**	**0.31**	**0.51**	0.11	−0.12
	Italian	0.30	**0.57**	0.30	**0.55**	0.28	−0.15
	Norwegian	0.23	**0.50**	0.21	**0.48**	0.15	−0.11
	Spanish	**0.34**	**0.52**	**0.35**	**0.51**	**0.31**	0.11
	Swedish	0.22	**0.47**	0.21	**0.44**	0.22	0.24
	*M*	0.30	0.57	0.30	0.53	0.26	0.03
	*Max*	0.36	0.71	0.37	0.59	0.34	0.24
	*Min*	0.22	0.47	0.21	0.44	0.11	−0.15
	*SD*	0.05	0.07	0.06	0.05	0.08	0.13
Emotions	Dutch	**0.33**	**0.71**	0.29	**0.71**	**0.36**	0.12
	English	0.20	**0.69**	0.20	**0.66**	**0.46**	0.09
	Finnish	**0.31**	**0.70**	**0.30**	**0.65**	**0.39**	0.11
	French	0.26	**0.71**	0.19	**0.68**	0.29	0.00
	German	0.28	**0.64**	0.23	**0.63**	0.09	−0.20
	Italian	0.23	**0.58**	0.22	**0.56**	0.21	−0.10
	Norwegian	**0.34**	**0.73**	0.27	**0.72**	**0.30**	−0.02
	Spanish	0.26	**0.44**	0.24	**0.43**	0.28	0.04
	Swedish	0.17	**0.67**	0.23	**0.61**	**0.45**	0.24
	*M*	0.26	0.65	0.24	0.63	0.31	0.03
	*Max*	0.34	0.73	0.30	0.72	0.46	0.24
	*Min*	0.17	0.44	0.19	0.43	0.09	−0.20
	*SD*	0.06	0.09	0.04	0.09	0.12	0.13
Physical	Dutch	**0.70**	**0.48**	**0.66**	**0.47**	**0.53**	0.14
	English	**0.64**	**0.44**	**0.65**	**0.45**	**0.57**	**0.28**
	Finnish	**0.66**	**0.44**	**0.62**	**0.43**	**0.58**	0.19
	French	**0.72**	**0.48**	**0.65**	**0.48**	**0.43**	0.07
	German	**0.73**	**0.35**	**0.69**	**0.42**	**0.56**	−0.04
	Italian	**0.56**	**0.55**	**0.55**	**0.54**	**0.47**	0.04
	Norwegian	**0.70**	**0.54**	**0.67**	**0.52**	**0.57**	0.14
	Spanish	**0.68**	**0.45**	**0.64**	**0.42**	**0.58**	0.12
	Swedish	**0.67**	0.26	**0.67**	0.19	**0.61**	**0.36**
	*M*	0.67	0.44	0.64	0.44	0.54	0.15
	*Max*	0.73	0.55	0.69	0.54	0.61	0.36
	*Min*	0.56	0.26	0.55	0.19	0.43	−0.04
	*SD*	0.05	0.09	0.04	0.10	0.06	0.12

Note. *M* = mean, *Max* = maximum, *Min* = minimum; *SD* = standard deviation; SF-36v2/SF-12v2—PCS = Physical Component Score; SF-36v2/SF-12v2—MCS = Mental Component Score; GCS = Glasgow Coma Scale; GOSE = Glasgow Outcome Scale—Extended. Values in **bold** represent an at least medium effect size (≥|0.30|), significant at α = 0.05.

**Table A4:** Convergent and divergent validity of the SF-36v2 scales.

		Convergent Validity	Discriminant Validity
Scale	Language/Value	GOSE	GCS
PF	Dutch	**0.45**	0.12
	English	**0.54**	0.22
	Finnish	**0.49**	0.08
	French	**0.36**	0.07
	German	**0.37**	−0.07
	Italian	**0.61**	0.13
	Norwegian	**0.52**	0.24
	Spanish	**0.52**	0.20
	Swedish	**0.43**	0.20
Total	*M*	0.48	0.13
	*Max*	0.61	0.24
	*Min*	0.36	−0.07
	*SD*	0.08	0.10
RP	Dutch	**0.63**	0.26
	English	**0.67**	**0.34**
	Finnish	**0.58**	0.19
	French	**0.48**	0.24
	German	0.24	0.06
	Italian	**0.67**	0.22
	Norwegian	**0.61**	0.24
	Spanish	**0.60**	0.22
	Swedish	**0.64**	**0.41**
Total	*M*	0.57	0.24
	*Max*	0.67	0.41
	*Min*	0.24	0.06
	*SD*	0.14	0.10
BP	Dutch	**0.39**	0.00
	English	**0.32**	0.04
	Finnish	**0.36**	−0.04
	French	**0.26**	0.06
	German	**0.43**	−0.08
	Italian	**0.37**	0.04
	Norwegian	**0.27**	−0.06
	Spanish	**0.44**	0.08
	Swedish	0.24	0.12
Total	*M*	0.34	0.02
	*Max*	0.44	0.12
	*Min*	0.24	−0.08
	*SD*	0.07	0.07
GH	Dutch	**0.33**	0.06
	English	**0.36**	0.11
	Finnish	**0.50**	0.03
	French	**0.39**	0.10
	German	0.26	0.00
	Italian	**0.42**	−0.02
	Norwegian	**0.39**	−0.03
	Spanish	**0.42**	0.10
	Swedish	**0.38**	0.20
Total	*M*	0.38	0.06
	*Max*	0.50	0.20
	*Min*	0.26	−0.03
	*SD*	0.07	0.08
VT	Dutch	**0.45**	0.16
	English	**0.43**	0.06
	Finnish	**0.52**	0.02
	French	**0.37**	0.08
	German	0.23	−0.08
	Italian	**0.43**	0.03
	Norwegian	**0.35**	−0.03
	Spanish	**0.38**	0.06
	Swedish	**0.47**	0.15
Total	*M*	0.40	0.05
	*Max*	0.52	0.16
	*Min*	0.23	−0.08
	*SD*	0.08	0.08
SF	Dutch	**0.55**	0.21
	English	**0.61**	0.25
	Finnish	**0.53**	0.14
	French	**0.49**	0.21
	German	0.24	−0.03
	Italian	**0.57**	0.13
	Norwegian	**0.57**	0.14
	Spanish	**0.54**	0.24
	Swedish	**0.50**	0.13
Total	*M*	0.51	0.16
	*Max*	0.61	0.25
	*Min*	0.24	−0.03
	*SD*	0.11	0.09
RE	Dutch	**0.43**	0.20
	English	**0.46**	0.11
	Finnish	**0.47**	0.11
	French	**0.37**	0.24
	German	0.13	−0.05
	Italian	**0.52**	0.13
	Norwegian	**0.37**	0.07
	Spanish	**0.48**	0.12
	Swedish	**0.46**	0.19
Total	*M*	0.41	0.12
	*Max*	0.52	0.24
	*Min*	0.13	−0.05
	*SD*	0.12	0.08
MH	Dutch	**0.38**	0.12
	English	**0.33**	0.04
	Finnish	**0.44**	0.12
	French	**0.34**	−0.04
	German	0.14	−0.07
	Italian	0.30	−0.12
	Norwegian	**0.31**	−0.09
	Spanish	**0.37**	0.10
	Swedish	**0.50**	0.10
Total	*M*	0.35	0.02
	*Max*	0.50	0.12
	*Min*	0.14	−0.12
	*SD*	0.10	0.10
PCS	Dutch	**0.49**	0.11
	English	**0.52**	0.22
	Finnish	**0.51**	0.06
	French	**0.37**	0.14
	German	**0.38**	−0.03
	Italian	**0.60**	0.17
	Norwegian	**0.53**	0.18
	Spanish	**0.53**	0.17
	Swedish	**0.46**	**0.30**
Total	*M*	0.49	0.15
	*Max*	0.60	0.30
	*Min*	0.37	−0.03
	*SD*	0.07	0.09
MCS	Dutch	**0.40**	0.19
	English	**0.42**	0.10
	Finnish	**0.46**	0.11
	French	**0.39**	0.15
	German	0.07	−0.10
	Italian	**0.37**	0.00
	Norwegian	**0.35**	−0.04
	Spanish	**0.37**	0.10
	Swedish	**0.50**	0.14
Total	*M*	0.37	0.07
	*Max*	0.50	0.19
	*Min*	0.07	−0.10
	*SD*	0.12	0.10

Note. *M* = mean, *Max* = maximum, *Min* = minimum; SD = standard deviation; PF = Physical functioning; BP = Bodily Pain; GH = General Health; VT = Vitality; SF = Social Functioning; RE = Role-Emotional; MH = Mental Health; PCS = Physical Component Score; MCS = Mental Component Score; GCS = Glasgow Coma Scale; GOSE = Glasgow Outcome Scale—Extended. No correlations with the Reported Health Transition (HT) scale reported because of the scale length (one item); values in **bold** represent an at least medium effect size (≥|0.30|), significant at α = 0.05.

## References

[1] Menon D.K., Schwab K., Wright D.W., Maas A.I. (2010). Position Statement: Definition of Traumatic Brain Injury. Arch. Phys. Med. Rehabil..

[2] Verhaeghe S., Defloor T., Grypdonck M. (2005). Stress and Coping among Families of Patients with Traumatic Brain Injury: A Review of the Literature. J. Clin. Nurs..

[3] Humphreys I., Wood R.L., Phillips C. (2013). Macey. The Costs of Traumatic Brain Injury: A Literature Review. Clin. Outcomes Res..

[4] Leibson C.L., Brown A.W., Long K.H., Ransom J.E., Mandrekar J., Osler T.M., Malec J.F. (2012). Medical Care Costs Associated with Traumatic Brain Injury over the Full Spectrum of Disease: A Controlled Population-Based Study. J. Neurotrauma.

[5] Cassidy J.D., Carroll L., Peloso P., Borg J., von Holst H., Holm L., Kraus J., Coronado V. (2004). Incidence, risk factors and prevention of mild traumatic brain injury: Results of the WHO collaborating centre task force on mild traumatic brain injury. J. Rehabil. Med..

[6] Dewan M.C., Rattani A., Gupta S., Baticulon R.E., Hung Y.-C., Punchak M., Agrawal A., Adeleye A.O., Shrime M.G., Rubiano A.M. (2019). Estimating the global incidence of traumatic brain injury. J. Neurosurg..

[7] Rabinowitz A.R., Levin H.S. (2014). Cognitive Sequelae of Traumatic Brain Injury. Psychiatr. Clin. N. Am..

[8] Bamdad M.J., Ryan L.M., Warden D.L. (2003). Functional Assessment of Executive Abilities Following Traumatic Brain Injury. Brain Inj..

[9] Hoofien D., Gilboa A., Vakil E., Donovick P.J. (2001). Traumatic Brain Injury (TBI) 10-20 Years Later: A Comprehensive Outcome Study of Psychiatric Symptomatology, Cognitive Abilities and Psychosocial Functioning. Brain Inj..

[10] Sasse N., Gibbons H., Wilson L., Martinez-Olivera R., Schmidt H., Hasselhorn M., von Wild K., von Steinbüchel N. (2013). Self-Awareness and Health-Related Quality of Life after Traumatic Brain Injury. J. Head Trauma Rehabil..

[11] Rauen K., Reichelt L., Probst P., Schäpers B., Müller F., Jahn K., Plesnila N. (2020). Quality of Life up to 10 Years after Traumatic Brain Injury: A Cross-Sectional Analysis. Health Qual. Life Outcomes.

[12] Bombardier C.H., Fann J.R., Temkin N.R., Esselman P.C., Barber J., Dikmen S.S. (2010). Rates of major depressive disorder and clinical outcomes following traumatic brain injury. JAMA.

[13] Bryant R.A., O’Donnell M.L., Creamer M., McFarlane A.C., Clark C.R., Silove D. (2010). The Psychiatric Sequelae of Traumatic Injury. AJP.

[14] Ponsford J., Draper K., Schönberger M. (2008). Functional Outcome 10 Years after Traumatic Brain Injury: Its Relationship with Demographic, Injury Severity, and Cognitive and Emotional Status. J. Inter. Neuropsych. Soc..

[15] Maas A.I.R., Menon D.K., Steyerberg E.W., Citerio G., Lecky F., Manley G.T., Hill S., Legrand V., Sorgner A. (2015). Collaborative European NeuroTrauma Effectiveness Research in Traumatic Brain Injury (CENTER-TBI): A Prospective Longitudinal Observational Study. Neurosurgery.

[16] Steyerberg E.W., Wiegers E., Sewalt C., Buki A., Citerio G., De Keyser V., Ercole A., Kunzmann K., Lanyon L., Lecky F. (2019). Case-Mix, Care Pathways, and Outcomes in Patients with Traumatic Brain Injury in CENTER-TBI: A European Prospective, Multicentre, Longitudinal, Cohort Study. Lancet Neurol..

[17] Gennarelli T.A., Wodzin E. (2006). AIS 2005: A Contemporary Injury Scale. Injury.

[18] Acquadro C., Conway K., Hareendran A., Aaronson N. (2008). Literature Review of Methods to Translate Health-Related Quality of Life Questionnaires for Use in Multinational Clinical Trials. Value Health.

[19] Acquadro C. (2012). Linguistic Validation Manual for Health Outcome Assessments.

[20] Steinbuechel N., Rauen K., Krenz U., Wu Y.-J., Covic A., Plass A.M., Cunitz K., Bockhop F., Polinder S., Wilson L. Translation and linguistic validation of outcome instruments for traumatic brain injury research and clinical practice: A step-by-step approach within the observational CENTER-TBI study. J. Clin. Med..

[21] NINDS Common Data Elements. http://www.commondataelements.ninds.nih.gov/.

[22] Wilde E.A., Whiteneck G.G., Bogner J., Bushnik T., Cifu D.X., Dikmen S., French L., Giacino J.T., Hart T., Malec J.F. (2010). Recommendations for the Use of Common Outcome Measures in Traumatic Brain Injury Research. Arch. Phys. Med. Rehabil..

[23] Spitzer R.L., Kroenke K., Williams J.B.W., Löwe B. (2006). A Brief Measure for Assessing Generalized Anxiety Disorder: The GAD-7. Arch. Intern. Med..

[24] Kroenke K., Spitzer R.L., Williams J.B.W. (2001). The PHQ-9: Validity of a Brief Depression Severity Measure. J. Gen. Intern. Med..

[25] Kroenke K., Spitzer R.L. (2002). The PHQ-9: A New Depression Diagnostic and Severity Measure. Psychiatr. Ann..

[26] Blevins C.A., Weathers F.W., Davis M.T., Witte T.K., Domino J.L. (2015). The Posttraumatic Stress Disorder Checklist for DSM-5 (PCL-5): Development and Initial Psychometric Evaluation. J. Trauma. Stress.

[27] American Psychiatric Association (2013). Diagnostic and Statistical Manual of Mental Disorders: DSM-5.

[28] Ashbaugh A.R., Houle-Johnson S., Herbert C., El-Hage W., Brunet A. (2016). Psychometric Validation of the English and French Versions of the Posttraumatic Stress Disorder Checklist for DSM-5 (PCL-5). PLoS ONE.

[29] Stein M.B., Jain S., Giacino J.T., Levin H., Dikmen S., Nelson L.D., Vassar M.J., Okonkwo D.O., Diaz-Arrastia R., Robertson C.S. (2019). Risk of Posttraumatic Stress Disorder and Major Depression in Civilian Patients After Mild Traumatic Brain Injury: A TRACK-TBI Study. Jama Psychiatry.

[30] King N.S., Crawford S., Wenden F.J., Moss N.E., Wade D.T. (1995). The Rivermead Post Concussion Symptoms Questionnaire: A Measure of Symptoms Commonly Experienced after Head Injury and Its Reliability. J. Neurol..

[31] Potter S., Leigh E., Wade D., Fleminger S. (2006). The Rivermead Post Concussion Symptoms Questionnaire: A Confirmatory Factor Analysis. J. Neurol..

[32] von Steinbuechel N., Wilson L., Gibbons H., Hawthorne G., Höfer S., Schmidt S., Bullinger M., Maas A., Neugebauer E., Powell J. (2010). Quality of Life after Brain Injury (QOLIBRI): Scale Development and Metric Properties. J. Neurotrauma.

[33] von Steinbuechel N., Wilson L., Gibbons H., Hawthorne G., Höfer S., Schmidt S., Bullinger M., Maas A., Neugebauer E., Powell J. (2010). Quality of Life after Brain Injury (QOLIBRI): Scale Validity and Correlates of Quality of Life. J. Neurotrauma.

[34] Wilson L., Marsden-Loftus I., Koskinen S., Bakx W., Bullinger M., Formisano R., Maas A., Neugebauer E., Powell J., Sarajuuri J. (2017). Interpreting Quality of Life after Brain Injury Scores: Cross-Walk with the Short Form-36. J. Neurotrauma.

[35] Gorbunova A., Zeldovich M., Voormolen D.C., Krenz U., Polinder S., Haagsma J.A., Hagmayer Y., Covic A., Real R.G.L., Asendorf T. (2020). Reference Values of the QOLIBRI from General Population Samples in the UK and The Netherlands. JCM.

[36] Siponkoski S., Wilson L., Steinbüchel N., Sarajuuri J., Koskinen S. (2013). Quality of Life after Traumatic Brain Injury: Finnish Experience of the QOLIBRI in Residential Rehabilitation. J. Rehabil. Med..

[37] Castaño-León A.M., Navarro-Main B., Gomez P.A., Gil A., Soler M.D., Lagares A., Bernabeu M., Steinbüchel N., Real R.G.L. (2018). Quality of Life After Brain Injury: Psychometric Properties of the Spanish Translation of the QoLIBRI. Eval. Health Prof..

[38] von Steinbuechel N., Wilson L., Gibbons H., Muehlan H., Schmidt H., Schmidt S., Sasse N., Koskinen S., Sarajuuri J., Höfer S. (2012). QOLIBRI Overall Scale: A Brief Index of Health-Related Quality of Life after Traumatic Brain Injury. J. Neurol. Neurosurg. Psychiatry.

[39] Wu Y.-J., Rauen K., Zeldovich M., Voormolen D.C., Gorbunova A., Covic A., Cunitz K., Plass A.M., Polinder S., Haagsma J.A. (2021). Reference Values and Psychometric Properties of the Quality of Life after Traumatic Brain Injury Overall Scale in Italy, the Netherlands, and the United Kingdom. Value Health.

[40] Ware J.E., Kosinski M., Bjorner J.B., Turner-Bowker D.M., Gandek B., Maruish M.E. (2007). User’s Manual for the 36v2 Health Survey.

[41] Maruish M.E., Maruish M., Kosinski M., Bjorner J.B., Gandek B., Turner-Bowker D.M., Ware J.E. (2011). User’s Manual for the SF-36v2 Health Survey.

[42] Ware J.E., Kosinski M., Turner-Bowker D.M., Gandek B. (2009). User’s Manual for the SF12v2 Health Survey.

[43] Fukuhara S., Ware J.E., Kosinski M., Wada S., Gandek B. (1998). Psychometric and Clinical Tests of Validity of the Japanese SF-36 Health Survey. J. Clin. Epidemiol..

[44] Optum The Optum® SF-36v2® Health Survey. https://www.optum.com/solutions/life-sciences/answer-research/patient-insights/sf-health-surveys/sf-36v2-health-survey.html.

[45] Wilson J.T.L., Pettigrew L.E.L., Teasdale G.M. (1998). Structured Interviews for the Glasgow Outcome Scale and the Extended Glasgow Outcome Scale: Guidelines for Their Use. J. Neurotrauma.

[46] Wilson L., Edwards P., Fiddes H., Stewart E., Teasdale G.M. (2002). Reliability of Postal Questionnaires for the Glasgow Outcome Scale. J. Neurotrauma.

[47] Kunzmann K., Wernisch L., Richardson S., Steyerberg E.W., Lingsma H., Ercole A., Maas A.I.R., Menon D., Wilson L. (2021). Imputation of Ordinal Outcomes: A Comparison of Approaches in Traumatic Brain Injury. J. Neurotrauma.

[48] Teasdale G., Jennett B. (1974). Assessment of Coma and Impaired Consciousness. A Practical Scale. Lancet.

[49] McDonald R.P. (1999). Test Theory: A Unified Treatment.

[50] Bulmer M.G. (1979). Principles of Statistics.

[51] Bujang M.A., Omar E.D., Baharum N.A. (2018). A Review on Sample Size Determination for Cronbach’s Alpha Test: A Simple Guide for Researchers. MJMS.

[52] Yurdugül H. (2008). Minimum Sample Size for Cronbach’s Coefficient Alpha. Hacettepe Üniversitesi Eğitim Fakültesi Dergisi.

[53] Hair J.F., Black W.C., Babin B.J., Anderson R.E. (2010). Multivariate Data Analysis.

[54] Fayers P., Machin D. (2013). Quality of Life the Assessment, Analysis and Interpretation of Patient-Reported Outcomes.

[55] Cho E., Kim S. (2015). Cronbach’s Coefficient Alpha: Well Known but Poorly Understood. Organ. Res. Methods.

[56] Cohen J. (1992). A Power Primer. Psychol. Bull..

[57] Cohen J. (1988). Statistical Power Analysis for the Behavioral Sciences.

[58] Terwee C.B., Bot S.D.M., de Boer M.R., van der Windt D.A.W.M., Knol D.L., Dekker J., Bouter L.M., de Vet H.C.W. (2007). Quality Criteria Were Proposed for Measurement Properties of Health Status Questionnaires. J. Clin. Epidemiol..

[59] Nelson L.D., Ranson J., Ferguson A.R., Giacino J., Okonkwo D.O., Valadka A.B., Manley G.T., McCrea M.A., the TRACK-TBI Investigators (2017). Validating Multi-Dimensional Outcome Assessment Using the Traumatic Brain Injury Common Data Elements: An Analysis of the TRACK-TBI Pilot Study Sample. J. Neurotrauma.

[60] Wolf E.J., Harrington K.M., Clark S.L., Miller M.W. (2013). Sample Size Requirements for Structural Equation Models: An Evaluation of Power, Bias, and Solution Propriety. Educ. Psychol. Meas..

[61] Brown T.A. (2015). Confirmatory Factor Analysis for Applied Research. Methodology in the Social Sciences.

[62] Bentler P.M. (1990). Comparative Fit Indexes in Structural Models. Psychol. Bull..

[63] Hu L., Bentler P.M. (1999). Cutoff Criteria for Fit Indexes in Covariance Structure Analysis: Conventional Criteria versus New Alternatives. Struct. Equ. Modeling A Multidiscip. J..

[64] Browne M., Cudeck R., Bollen K.A., Long S.J. (1993). Alternative ways of assessing model fit. Testing Structural Equation.

[65] Revelle W. (2020). Psych: Procedures for Psychological, Psychometric, and Personality Research.

[66] Rosseel Y. (2012). Lavaan: An R Package for Structural Equation Modeling. J. Stat. Soft..

[67] R Core Team (2020). R: A Language and Environment for Statistical Computing.

[68] Hart T., Fann J.R., Chervoneva I., Juengst S.B., Rosenthal J.A., Krellman J.W., Dreer L.E., Kroenke K. (2016). Prevalence, Risk Factors, and Correlates of Anxiety at 1 Year After Moderate to Severe Traumatic Brain Injury. Arch. Phys. Med. Rehabil..

[69] Donders J., Darland K. (2017). Psychometric Properties and Correlates of the PHQ-2 and PHQ-9 after Traumatic Brain Injury. Brain Inj..

[70] Fann J.R., Bombardier C.H., Dikmen S., Esselman P., Warms C.A., Pelzer E., Rau H., Temkin N. (2005). Validity of the Patient Health Questionnaire-9 in Assessing Depression Following Traumatic Brain Injury. J. Head Trauma Rehabil..

[71] Wortmann J.H., Jordan A.H., Weathers F.W., Resick P.A., Dondanville K.A., Hall-Clark B., Foa E.B., Young-McCaughan S., Yarvis J.S., Hembree E.A. (2016). Psychometric Analysis of the PTSD Checklist-5 (PCL-5) among Treatment-Seeking Military Service Members. Psychol. Assess..

[72] Eyres S., Carey A., Gilworth G., Neumann V., Tennant A. (2005). Construct Validity and Reliability of the Rivermead Post-Concussion Symptoms Questionnaire. Clin. Rehabil..

[73] Smith-Seemiller L., Fow N.R., Kant R., Franzen M.D. (2003). Presence of Post-Concussion Syndrome Symptoms in Patients with Chronic Pain vs Mild Traumatic Brain Injury. Brain Inj..

[74] Miller M.W., Wolf E.J., Kilpatrick D., Resnick H., Marx B.P., Holowka D.W., Keane T.M., Rosen R.C., Friedman M.J. (2013). The Prevalence and Latent Structure of Proposed DSM-5 Posttraumatic Stress Disorder Symptoms in U.S. National and Veteran Samples. Psychol. Trauma Theory Res. Pract. Policy.

[75] Biehn T.L., Elhai J.D., Seligman L.D., Tamburrino M., Armour C., Forbes D. (2013). Underlying Dimensions of DSM-5 Posttraumatic Stress Disorder and Major Depressive Disorder Symptoms. Psychol. Inj. Law.

[76] Teymoori A., Gorbunova A., Haghish F.E., Real R., Zeldovich M., Wu Y.-J., Polinder S., Asendorf T., Menon D., CENTER-TBI Investigators and Participants CENTER-TBI Investigators and Participants (2020). Factorial Structure and Validity of Depression (PHQ-9) and Anxiety (GAD-7) Scales after Traumatic Brain Injury. JCM.

[77] Geier T.J., Hunt J.C., Hanson J.L., Heyrman K., Larsen S.E., Brasel K.J., deRoon-Cassini T.A. (2020). Validation of Abbreviated Four- and Eight-Item Versions of the PTSD Checklist for *DSM-5* in a Traumatically Injured Sample: Abbreviated PCL-5 Validation in Traumatic Injury. J. Trauma. Stress.

[78] von Steinbuechel N., Covic A., Polinder S., Kohlmann T., Cepulyte U., Poinstingl H., Backhaus J., Bakx W., Bullinger M., Christensen A.-L. (2016). Assessment of Health-Related Quality of Life after TBI: Comparison of a Disease-Specific (QOLIBRI) with a Generic (SF-36) Instrument. Behav. Neurol..

[79] Zahniser E., Nelson L.D., Dikmen S.S., Machamer J.E., Stein M.B., Yuh E., Manley G.T., Temkin N.R. (2019). TRACK-TBI Investigators the Temporal Relationship of Mental Health Problems and Functional Limitations Following MTBI: A TRACK-TBI and TED Study. J. Neurotrauma.

[80] Polinder S., Cnossen M.C., Real R.G.L., Covic A., Gorbunova A., Voormolen D.C., Master C.L., Haagsma J.A., Diaz-Arrastia R., von Steinbuechel N. (2018). A Multidimensional Approach to Post-Concussion Symptoms in Mild Traumatic Brain Injury. Front. Neurol..

[81] Von Steinbüchel N., Meeuwsen M., Zeldovich M., Vester J.C., Maas A., Koskinen S., Covic A. (2020). Differences in Health-Related Quality of Life after Traumatic Brain Injury between Varying Patient Groups: Sensitivity of a Disease-Specific (QOLIBRI) and a Generic (SF-36) Instrument. J. Neurotrauma.

[82] Rockwood T.H., Sangster R.L., Dillman D.A. (1997). The Effect of Response Categories on Questionnaire Answers: Context and Mode Effects. Sociol. Methods Res..

[83] Maydeu-Olivares A., Kramp U., García-Forero C., Gallardo-Pujol D., Coffman D. (2009). The Effect of Varying the Number of Response Alternatives in Rating Scales: Experimental Evidence from Intra-Individual Effects. Behav. Res. Methods.

[84] Maydeu-Olivares A., Fairchild A.J., Hall A.G. (2017). Goodness of Fit in Item Factor Analysis: Effect of the Number of Response Alternatives. Struct. Equ. Modeling Multidiscip. J..

[85] Teymoori A., Real R., Gorbunova A., Haghish E.F., Andelic N., Wilson L., Asendorf T., Menon D., von Steinbüchel N. (2020). Measurement Invariance of Assessments of Depression (PHQ-9) and Anxiety (GAD-7) across Sex, Strata and Linguistic Backgrounds in a European-Wide Sample of Patients after Traumatic Brain Injury. J. Affect. Disord..

